# Supplementary material for: Multi-task Bayesian model combining FDG-PET/CT imaging and clinical data for interpretable high-grade prostate cancer prognosis
Source: Sci Rep. 2024 Nov 6;14:26928. doi: 10.1038/s41598-024-77498-0 (PMC11541986; doi:10.1038/s41598-024-77498-0)
Supplement: Supplementary file 1 — Supplementary Information. [file 41598_2024_77498_MOESM1_ESM.pdf]

Multi-task Bayesian Model Combining FDG-PET/CT Imaging and Clinical Data for Interpretable High-Grade Prostate Cancer Prognosis

1 Cohort description

1.1 Descriptive analysis of clinical features

Supplementary Table 1 Descriptive analysis of the clinical features for the LNI task on the *full dataset*, the *learning set*, and the *holdout set*. The *p*-values are computed using the Mann-Whitney U test [1] for continuous clinical features (age and psa) and the χ^2 test [2] for categorical features (clinical stage, global Gleason score, primary Gleason score, and secondary Gleason score) using `scipy` [3] Python library.

	Full dataset				Learning set				Holdout set			
Feature	All $n = 295$ 100.0 %	Negative $n = 209$ 70.8 %	Positive $n = 86$ 29.2 %	*p*-value	All $n = 250$ 100.0 %	Negative $n = 177$ 70.8 %	Positive $n = 73$ 29.2 %	*p*-value	All $n = 45$ 100.0 %	Negative $n = 32$ 71.1 %	Positive $n = 13$ 28.9 %	*p*-value
Age [years]				0.8035				0.7697				0.9600
Mean (Median)	65.4 (66.0)	65.4 (66.0)	65.3 (66.0)		65.6 (66.0)	65.6 (66.0)	65.4 (66.0)		64.4 (66.0)	64.4 (65.5)	64.5 (66.0)	
Min - Max	48.0 - 80.0	48.0 - 80.0	48.0 - 78.0		48.0 - 80.0	48.0 - 80.0	48.0 - 78.0		50.0 - 76.0	50.0 - 76.0	53.0 - 75.0	
PSA [ng/ml]				0.0572				0.0854				0.5903
Mean (Median)	11.0 (7.4)	9.9 (7.1)	13.5 (8.1)		11.5 (7.4)	10.3 (7.1)	14.4 (8.2)		8.0 (7.4)	7.9 (7.0)	8.3 (7.4)	
Min - Max	1.1 - 155.3	1.1 - 155.3	1.1 - 110.0		1.1 - 155.3	1.4 - 155.3	1.1 - 110.0		1.1 - 17.7	1.1 - 17.7	5.5 - 16.7	
Clinical stage				< 0.0001				< 0.0001				0.5913
T1-T2	230 (88.1)	176 (95.1)	54 (71.1)		196 (87.9)	150 (95.5)	46 (69.7)		34 (89.5)	26 (92.9)	8 (80.0)	
T3a	31 (11.9)	9 (4.9)	22 (28.9)		27 (12.1)	7 (4.5)	20 (30.3)		4 (10.5)	2 (7.1)	2 (20.0)	
Global Gleason				0.0020				0.0031				0.2363
8	188 (63.7)	146 (69.9)	42 (48.8)		160 (64.0)	124 (70.1)	36 (49.3)		28 (62.2)	22 (68.8)	6 (46.2)	
9	106 (35.9)	62 (29.7)	44 (51.2)		90 (36.0)	53 (29.9)	37 (50.7)		16 (35.6)	9 (28.1)	7 (53.8)	
10	1 (0.3)	1 (0.5)	0 (0.0)		0 (0.0)	0 (0.0)	0 (0.0)		1 (2.2)	1 (3.1)	0 (0.0)	
Primary Gleason				0.3240				0.4349				0.5732
3	16 (5.4)	13 (6.2)	3 (3.5)		14 (5.6)	11 (6.2)	3 (4.1)		2 (4.4)	2 (6.2)	0 (0.0)	
4	268 (90.8)	190 (90.9)	78 (90.7)		230 (92.0)	163 (92.1)	67 (91.8)		38 (84.4)	27 (84.4)	11 (84.6)	
5	11 (3.7)	6 (2.9)	5 (5.8)		6 (2.4)	3 (1.7)	3 (4.1)		5 (11.1)	3 (9.4)	2 (15.4)	
Secondary Gleason				0.0324				0.0247				0.5486
3	2 (0.7)	1 (0.5)	1 (1.2)		0 (0.0)	0 (0.0)	0 (0.0)		2 (4.4)	1 (3.1)	1 (7.7)	
4	178 (60.3)	136 (65.1)	42 (48.8)		152 (60.8)	116 (65.5)	36 (49.3)		26 (57.8)	20 (62.5)	6 (46.2)	
5	115 (39.0)	72 (34.4)	43 (50.0)		98 (39.2)	61 (34.5)	37 (50.7)		17 (37.8)	11 (34.4)	6 (46.2)	

Supplementary Table 2 Descriptive analysis of the clinical features for the BCR-FS task on the *full dataset*, the *learning set*, and the *holdout set*. The p-values are computed using the Mann-Whitney U test [1] for continuous clinical features (age and psa) and the χ^2 test [2] for categorical features (clinical stage, global Gleason score, primary Gleason score, and secondary Gleason score) using `scipy` [3] Python library.

	Full dataset				Learning set				Holdout set			
Feature	All<br>$n = 289$<br>100.0 %	Negative<br>$n = 129$<br>44.6 %	Positive<br>$n = 160$<br>55.4 %	p-value	All<br>$n = 245$<br>100.0 %	Negative<br>$n = 112$<br>45.7 %	Positive<br>$n = 133$<br>54.3 %	p-value	All<br>$n = 44$<br>100.0 %	Negative<br>$n = 17$<br>38.6 %	Positive<br>$n = 27$<br>61.4 %	p-value
Age [years]				0.1391				0.1541				0.7444
Mean (Median)	65.4 (66.0)	66.1 (67.0)	64.9 (65.0)		65.6 (66.0)	66.2 (67.0)	65.0 (65.0)		64.5 (66.0)	65.1 (66.0)	64.1 (66.0)	
Min - Max	48.0 - 80.0	48.0 - 80.0	48.0 - 78.0		48.0 - 80.0	48.0 - 80.0	48.0 - 78.0		50.0 - 76.0	53.0 - 76.0	50.0 - 75.0	
PSA [ng/ml]				0.0271				0.0170				0.885
Mean (Median)	11.0 (7.4)	8.5 (7.0)	13.0 (7.9)		11.6 (7.4)	8.6 (7.0)	14.0 (8.2)		7.9 (7.3)	8.1 (7.7)	7.8 (7.2)	
Min - Max	1.1 - 155.3	1.1 - 48.0	1.4 - 155.3		1.1 - 155.3	1.1 - 48.0	1.4 - 155.3		1.1 - 17.7	1.1 - 17.7	2.4 - 16.7	
Clinical stage				0.1518				0.1921				0.9320
T1-T2	225 (87.9)	100 (91.7)	125 (85.0)		191 (87.6)	86 (91.5)	105 (84.7)		34 (89.5)	14 (93.3)	20 (87.0)	
T3a	31 (12.1)	9 (8.3)	22 (15.0)		27 (12.4)	8 (8.5)	19 (15.3)		4 (10.5)	1 (6.7)	3 (13.0)	
Global Gleason				0.0395				0.0291				1.0000
8	184 (63.7)	91 (70.5)	93 (58.1)		156 (63.7)	80 (71.4)	76 (57.1)		28 (63.6)	11 (64.7)	17 (63.0)	
9	105 (36.3)	38 (29.5)	67 (41.9)		89 (36.3)	32 (28.6)	57 (42.9)		16 (36.4)	6 (35.3)	10 (37.0)	
10	0 (0.0)	0 (0.0)	0 (0.0)		0 (0.0)	0 (0.0)	0 (0.0)		0 (0.0)	0 (0.0)	0 (0.0)	
Primary Gleason				0.0944				0.3045				0.1702
3	16 (5.5)	11 (8.5)	5 (3.1)		14 (5.7)	9 (8.0)	5 (3.8)		2 (4.5)	2 (11.8)	0 (0.0)	
4	263 (91.0)	115 (89.1)	148 (92.5)		225 (91.8)	101 (90.2)	124 (93.2)		38 (86.4)	14 (82.4)	24 (88.9)	
5	10 (3.5)	3 (2.3)	7 (4.4)		6 (2.4)	2 (1.8)	4 (3.0)		4 (9.1)	1 (5.9)	3 (11.1)	
Secondary Gleason				0.2254				0.2041				0.4877
3	2 (0.7)	0 (0.0)	2 (1.2)		0 (0.0)	0 (0.0)	0 (0.0)		2 (4.5)	0 (0.0)	2 (7.4)	
4	174 (60.2)	83 (64.3)	91 (56.9)		148 (60.4)	73 (65.2)	75 (56.4)		26 (59.1)	10 (58.8)	16 (59.3)	
5	113 (39.1)	46 (35.7)	67 (41.9)		97 (39.6)	39 (34.8)	58 (43.6)		16 (36.4)	7 (41.2)	9 (33.3)	

Supplementary Table 3 Descriptive analysis of the clinical features for the MFS task on the *full dataset*, the *learning set*, and the *holdout set*. The p-values are computed using the Mann-Whitney U test [1] for continuous clinical features (age and psa) and the χ^2 test [2] for categorical features (clinical stage, global Gleason score, primary Gleason score, and secondary Gleason score) using `scipy` [3] Python library.

	Full dataset				Learning set				Holdout set			
Feature	All<br>$n = 157$<br>100.0 %	Negative<br>$n = 119$<br>75.8 %	Positive<br>$n = 38$<br>24.2 %	p-value	All<br>$n = 131$<br>100.0 %	Negative<br>$n = 99$<br>75.6 %	Positive<br>$n = 32$<br>24.4 %	p-value	All<br>$n = 26$<br>100.0 %	Negative<br>$n = 20$<br>76.9 %	Positive<br>$n = 6$<br>23.1 %	p-value
Age [years]				0.0312				0.0357				0.6038
Mean (Median)	64.7 (65.0)	65.4 (66.0)	62.8 (62.5)		64.7 (65.0)	65.4 (66.0)	62.7 (62.5)		64.7 (66.0)	65.2 (66.5)	63.3 (63.5)	
Min - Max	48.0 - 78.0	48.0 - 78.0	48.0 - 77.0		48.0 - 78.0	48.0 - 78.0	48.0 - 77.0		53.0 - 75.0	53.0 - 75.0	54.0 - 73.0	
PSA [ng/ml]				0.0255				0.0277				0.9272
Mean (Median)	12.5 (7.8)	12.2 (7.4)	13.4 (10.0)		13.4 (8.2)	13.1 (7.5)	14.4 (10.3)		7.8 (7.3)	7.7 (7.3)	8.0 (6.9)	
Min - Max	1.1 - 155.3	1.1 - 155.3	2.6 - 85.3		1.1 - 155.3	1.1 - 155.3	2.6 - 85.3		3.0 - 16.7	3.0 - 16.7	5.3 - 13.3	
Clinical stage				0.2874				0.3531				1.0000
T1-T2	119 (83.8)	93 (86.1)	26 (76.5)		99 (83.2)	77 (85.6)	22 (75.9)		20 (87.0)	16 (88.9)	4 (80.0)	
T3a	23 (16.2)	15 (13.9)	8 (23.5)		20 (16.8)	13 (14.4)	7 (24.1)		3 (13.0)	2 (11.1)	1 (20.0)	
Global Gleason				0.0020				0.0076				0.2540
8	101 (64.3)	85 (71.4)	16 (42.1)		85 (64.9)	71 (71.7)	14 (43.8)		16 (61.5)	14 (70.0)	2 (33.3)	
9	56 (35.7)	34 (28.6)	22 (57.9)		46 (35.1)	28 (28.3)	18 (56.2)		10 (38.5)	6 (30.0)	4 (66.7)	
10	0 (0.0)	0 (0.0)	0 (0.0)		0 (0.0)	0 (0.0)	0 (0.0)		0 (0.0)	0 (0.0)	0 (0.0)	
Primary Gleason				0.1414				0.2097				0.5656
3	7 (4.5)	6 (5.0)	1 (2.6)		6 (4.6)	5 (5.1)	1 (3.1)		1 (3.8)	1 (5.0)	0 (0.0)	
4	145 (92.4)	111 (93.3)	34 (89.5)		122 (93.1)	93 (93.9)	29 (90.6)		23 (88.5)	18 (90.0)	5 (83.3)	
5	5 (3.2)	2 (1.7)	3 (7.9)		3 (2.3)	1 (1.0)	2 (6.2)		2 (7.7)	1 (5.0)	1 (16.7)	
Secondary Gleason				0.0809				0.0569				0.7225
3	1 (0.6)	1 (0.8)	0 (0.0)		0 (0.0)	0 (0.0)	0 (0.0)		1 (3.8)	1 (5.0)	0 (0.0)	
4	97 (61.8)	79 (66.4)	18 (47.4)		82 (62.6)	67 (67.7)	15 (46.9)		15 (57.7)	12 (60.0)	3 (50.0)	
5	59 (37.6)	39 (32.8)	20 (52.6)		49 (37.4)	32 (32.3)	17 (53.1)		10 (38.5)	7 (35.0)	3 (50.0)	

Supplementary Table 4 Descriptive analysis of the clinical features for the dADT-FS task on the *full dataset*, the *learning set*, and the *holdout set*. The p-values are computed using the Mann-Whitney U test [1] for continuous clinical features (age and psa) and the χ^2 test [2] for categorical features (clinical stage, global Gleason score, primary Gleason score, and secondary Gleason score) using `scipy` [3] Python library.

	Full dataset				Learning set				Holdout set			
Feature	All<br>$n = 282$<br>100.0 %	Negative<br>$n = 210$<br>74.5 %	Positive<br>$n = 72$<br>25.5 %	p-value	All<br>$n = 239$<br>100.0 %	Negative<br>$n = 173$<br>72.4 %	Positive<br>$n = 66$<br>27.6 %	p-value	All<br>$n = 43$<br>100.0 %	Negative<br>$n = 37$<br>86.0 %	Positive<br>$n = 6$<br>14.0 %	p-value
Age [years]				0.8754				0.9975				0.4716
Mean (Median)	65.2 (65.0)	65.3 (66.0)	65.2 (65.0)		65.4 (65.0)	65.4 (66.0)	65.4 (65.0)		64.3 (65.0)	64.6 (66.0)	62.7 (62.5)	
Min - Max	48.0 - 80.0	48.0 - 80.0	48.0 - 78.0		48.0 - 80.0	48.0 - 80.0	48.0 - 78.0		50.0 - 76.0	50.0 - 76.0	54.0 - 73.0	
PSA [ng/ml]				0.0008				0.0012				0.5749
Mean (Median)	10.7 (7.3)	9.4 (6.9)	14.5 (9.0)		11.2 (7.3)	9.7 (6.9)	15.1 (9.2)		7.9 (7.4)	7.8 (7.2)	8.4 (8.1)	
Min - Max	1.1 - 155.3	1.1 - 155.3	1.8 - 110.0		1.1 - 155.3	1.1 - 155.3	1.8 - 110.0		1.1 - 17.7	1.1 - 17.7	5.3 - 13.3	
Clinical stage				0.0198				0.0205				1.0000
T1-T2	221 (89.1)	166 (92.2)	55 (80.9)		189 (89.2)	139 (92.7)	50 (80.6)		32 (88.9)	27 (90.0)	5 (83.3)	
T3a	27 (10.9)	14 (7.8)	13 (19.1)		23 (10.8)	11 (7.3)	12 (19.4)		4 (11.1)	3 (10.0)	1 (16.7)	
Global Gleason				0.0021				0.0012				0.6701
8	180 (63.8)	146 (69.5)	34 (47.2)		153 (64.0)	122 (70.5)	31 (47.0)		27 (62.8)	24 (64.9)	3 (50.0)	
9	101 (35.8)	63 (30.0)	38 (52.8)		86 (36.0)	51 (29.5)	35 (53.0)		15 (34.9)	12 (32.4)	3 (50.0)	
10	1 (0.4)	1 (0.5)	0 (0.0)		0 (0.0)	0 (0.0)	0 (0.0)		1 (2.3)	1 (2.7)	0 (0.0)	
Primary Gleason				0.1675				0.2341				0.1834
3	16 (5.7)	13 (6.2)	3 (4.2)		14 (5.9)	11 (6.4)	3 (4.5)		2 (4.7)	2 (5.4)	0 (0.0)	
4	256 (90.8)	192 (91.4)	64 (88.9)		220 (92.1)	160 (92.5)	60 (90.9)		36 (83.7)	32 (86.5)	4 (66.7)	
5	10 (3.5)	5 (2.4)	5 (6.9)		5 (2.1)	2 (1.2)	3 (4.5)		5 (11.6)	3 (8.1)	2 (33.3)	
Secondary Gleason				0.0336				0.0145				0.3211
3	2 (0.7)	1 (0.5)	1 (1.4)		0 (0.0)	0 (0.0)	0 (0.0)		2 (4.7)	1 (2.7)	1 (16.7)	
4	169 (59.9)	135 (64.3)	34 (47.2)		144 (60.3)	113 (65.3)	31 (47.0)		25 (58.1)	22 (59.5)	3 (50.0)	
5	111 (39.4)	74 (35.2)	37 (51.4)		95 (39.7)	60 (34.7)	35 (53.0)		16 (37.2)	14 (37.8)	2 (33.3)	

Supplementary Table 5 Descriptive analysis of the clinical features for the CRPC-FS task on the *full dataset*, the *learning set*, and the *holdout set*. The p-values are computed using the Mann-Whitney U test [1] for continuous clinical features (age and psa) and the χ^2 test [2] for categorical features (clinical stage, global Gleason score, primary Gleason score, and secondary Gleason score) using `scipy` [3] Python library.

	Full dataset				Learning set				Holdout set			
Feature	All<br>$n = 290$<br>100.0 %	Negative<br>$n = 267$<br>92.1 %	Positive<br>$n = 23$<br>7.9 %	p-value	All<br>$n = 246$<br>100.0 %	Negative<br>$n = 227$<br>92.3 %	Positive<br>$n = 19$<br>7.7 %	p-value	All<br>$n = 44$<br>100.0 %	Negative<br>$n = 33$<br>90.9 %	Positive<br>$n = 4$<br>9.1 %	p-value
Age [years]				0.0383				0.0337				0.7904
Mean (Median)	65.4 (66.0)	65.6 (66.0)	62.7 (62.0)		65.6 (66.0)	65.8 (66.0)	62.5 (62.0)		64.4 (66.0)	64.5 (66.0)	63.5 (63.5)	
Min - Max	48.0 - 80.0	48.0 - 80.0	48.0 - 74.0		48.0 - 80.0	48.0 - 80.0	48.0 - 74.0		50.0 - 76.0	50.0 - 76.0	54.0 - 73.0	
PSA [ng/ml]				0.0032				0.0044				0.4142
Mean (Median)	10.7 (7.4)	10.3 (7.1)	15.3 (10.4)		11.1 (7.4)	10.7 (7.1)	16.6 (11.0)		8.0 (7.3)	7.9 (7.0)	9.1 (8.7)	
Min - Max	1.1 - 155.3	1.1 - 155.3	2.6 - 85.3		1.1 - 155.3	1.1 - 155.3	2.6 - 85.3		1.1 - 17.7	1.1 - 17.7	5.6 - 13.3	
Clinical stage				0.1347				0.2290				0.9083
T1-T2	228 (89.1)	211 (90.2)	17 (77.3)		195 (89.0)	181 (90.0)	14 (77.8)		33 (89.2)	30 (90.9)	3 (75.0)	
T3a	28 (10.9)	23 (9.8)	5 (22.7)		24 (11.0)	20 (10.0)	4 (22.2)		4 (10.8)	3 (9.1)	1 (25.0)	
Global Gleason				0.4467				0.7260				0.2400
8	185 (63.8)	173 (64.8)	12 (52.2)		158 (64.2)	147 (64.8)	11 (57.9)		27 (61.4)	26 (65.0)	1 (25.0)	
9	104 (35.9)	93 (34.8)	11 (47.8)		88 (35.8)	80 (35.2)	8 (42.1)		16 (36.4)	13 (32.5)	3 (75.0)	
10	1 (0.3)	1 (0.4)	0 (0.0)		0 (0.0)	0 (0.0)	0 (0.0)		1 (2.3)	1 (2.5)	0 (0.0)	
Primary Gleason				0.2034				0.3591				0.4745
3	16 (5.5)	14 (5.2)	2 (8.7)		14 (5.7)	12 (5.3)	2 (10.5)		2 (4.5)	2 (5.0)	0 (0.0)	
4	265 (91.4)	246 (92.1)	19 (82.6)		227 (92.3)	211 (93.0)	16 (84.2)		38 (86.4)	35 (87.5)	3 (75.0)	
5	9 (3.1)	7 (2.6)	2 (8.7)		5 (2.0)	4 (1.8)	1 (5.3)		4 (9.1)	3 (7.5)	1 (25.0)	
Secondary Gleason				0.6625				0.6223				0.8570
3	1 (0.3)	1 (0.4)	0 (0.0)		0 (0.0)	0 (0.0)	0 (0.0)		1 (2.3)	1 (2.5)	0 (0.0)	
4	175 (60.3)	163 (61.0)	12 (52.2)		149 (60.6)	139 (61.2)	10 (52.6)		26 (59.1)	24 (60.0)	2 (50.0)	
5	114 (39.3)	103 (38.6)	11 (47.8)		97 (39.4)	88 (38.8)	9 (47.4)		17 (38.6)	15 (37.5)	2 (50.0)	

Supplementary Table 6 Descriptive analysis of the clinical features for the PCSS task on the *full dataset*, the *learning set*, and the *holdout set*. The p-values are computed using the Mann-Whitney U test [1] for continuous clinical features (age and psa) and the χ^2 test [2] for categorical features (clinical stage, global Gleason score, primary Gleason score, and secondary Gleason score) using `scipy` [3] Python library.

	Full dataset				Learning set				Holdout set			
Feature	All $n = 295$ 100.0 %	Negative $n = 284$ 96.3 %	Positive $n = 11$ 3.7 %	p-value	All $n = 250$ 100.0 %	Negative $n = 241$ 96.4 %	Positive $n = 9$ 3.6 %	p-value	All $n = 45$ 100.0 %	Negative $n = 43$ 95.6 %	Positive $n = 2$ 4.4 %	p-value
Age [years]				0.7197				0.9719				0.3774
Mean (Median)	65.4 (66.0)	65.4 (66.0)	64.3 (66.0)		65.6 (66.0)	65.6 (66.0)	65.2 (67.0)		64.4 (66.0)	64.6 (66.0)	60.0 (60.0)	
Min - Max	48.0 - 80.0	48.0 - 80.0	48.0 - 77.0		48.0 - 80.0	48.0 - 80.0	48.0 - 77.0		50.0 - 76.0	50.0 - 76.0	54.0 - 66.0	
PSA [ng/ml]				0.0494				0.0879				0.3082
Mean (Median)	11.0 (7.4)	10.7 (7.2)	17.3 (10.0)		11.5 (7.4)	11.2 (7.3)	18.9 (10.0)		8.0 (7.4)	7.9 (7.2)	10.4 (10.4)	
Min - Max	1.1 - 155.3	1.1 - 155.3	2.6 - 85.3		1.1 - 155.3	1.1 - 155.3	2.6 - 85.3		1.1 - 17.7	1.1 - 17.7	7.4 - 13.3	
Clinical stage				0.0367				0.1413				0.4932
T1-T2	230 (88.1)	223 (89.2)	7 (63.6)		196 (87.9)	190 (88.8)	6 (66.7)		34 (89.5)	33 (91.7)	1 (50.0)	
T3a	31 (11.9)	27 (10.8)	4 (36.4)		27 (12.1)	24 (11.2)	3 (33.3)		4 (10.5)	3 (8.3)	1 (50.0)	
Global Gleason				0.7873				0.8541				0.8960
8	188 (63.7)	182 (64.1)	6 (54.5)		160 (64.0)	155 (64.3)	5 (55.6)		28 (62.2)	27 (62.8)	1 (50.0)	
9	106 (35.9)	101 (35.6)	5 (45.5)		90 (36.0)	86 (35.7)	4 (44.4)		16 (35.6)	15 (34.9)	1 (50.0)	
10	1 (0.3)	1 (0.4)	0 (0.0)		0 (0.0)	0 (0.0)	0 (0.0)		1 (2.2)	1 (2.3)	0 (0.0)	
Primary Gleason				0.5308				0.6906				0.1983
3	16 (5.4)	15 (5.3)	1 (9.1)		14 (5.6)	13 (5.4)	1 (11.1)		2 (4.4)	2 (4.7)	0 (0.0)	
4	268 (90.8)	259 (91.2)	9 (81.8)		230 (92.0)	222 (92.1)	8 (88.9)		38 (84.4)	37 (86.0)	1 (50.0)	
5	11 (3.7)	10 (3.5)	1 (9.1)		6 (2.4)	6 (2.5)	0 (0.0)		5 (11.1)	4 (9.3)	1 (50.0)	
Secondary Gleason				0.8764				0.4991				0.4654
3	2 (0.7)	2 (0.7)	0 (0.0)		0 (0.0)	0 (0.0)	0 (0.0)		2 (4.4)	2 (4.7)	0 (0.0)	
4	178 (60.3)	172 (60.6)	6 (54.5)		152 (60.8)	148 (61.4)	4 (44.4)		26 (57.8)	24 (55.8)	2 (100.0)	
5	115 (39.0)	110 (38.7)	5 (45.5)		98 (39.2)	93 (38.6)	5 (55.6)		17 (37.8)	17 (39.5)	0 (0.0)	

1.2 Descriptive analysis of outcomes

Supplementary Table 7 Survival time analysis for each task on the *full dataset*, the *learning set*, and the *holdout set*, with all durations measured in months from the date of radical prostatectomy (RP). Survival time, also known as failure time, refers to the duration of time between RP and the particular event (task) of interest. For instance, the median survival time is obtained by calculating the median of the observed times of patients who did experience failure.

	Full dataset			Learning set			Holdout set		
Task	Mean (Median)	Min-Max	Std	Mean (Median)	Min-Max	Std	Mean (Median)	Min-Max	Std
BCR-FS	20.39 (9.05)	0.89 - 106.02	25.18	19.52 (7.59)	0.89 - 101.89	24.87	24.71 (14.88)	1.94 - 106.02	26.72
MFS	35.19 (26.68)	0.03 - 101.85	27.49	34.83 (26.68)	0.03 - 101.85	26.90	37.05 (23.23)	5.32 - 92.06	33.13
dADT-FS	31.06 (18.69)	2.50 - 103.70	28.51	31.22 (19.30)	2.50 - 103.70	27.99	29.31 (11.19)	3.84 - 95.74	36.80
CRPC-FS	36.93 (34.79)	11.27 - 87.03	21.77	39.06 (35.68)	12.39 - 87.03	21.46	26.79 (17.17)	11.27 - 61.57	23.35
PCSS	60.51 (57.30)	23.43 - 102.57	26.40	61.09 (57.29)	23.43 - 102.57	28.96	57.92 (57.92)	46.85 - 68.99	15.66

Supplementary Table 8 Median follow-up time in the *full dataset*, the *learning set*, and the *holdout set*, with all durations measured in months from the date of radical prostatectomy (RP). Three methods are used to calculate the median follow-up time [4–6]: considering all patients in the study, regardless of censoring or failure (T_{obs}); using only patients who did not experience failure (T_{cens}); or generating a reverse Kaplan-Meier curve, where censoring and failure are swapped, and using the point at 50% of this curve as the median follow-up ($T_{\text{R-KM}}$).

	Full dataset			Learning set			Holdout set		
Task	T_{obs}	T_{cens}	$T_{\text{R-KM}}$	T_{obs}	T_{cens}	$T_{\text{R-KM}}$	T_{obs}	T_{cens}	$T_{\text{R-KM}}$
BCR-FS	20.60	38.11	66.86	20.60	41.05	66.86	20.42	24.15	55.29
MFS	55.33	84.80	87.29	56.57	86.64	88.71	47.28	66.53	82.83
dADT-FS	49.31	60.29	73.49	49.53	66.86	75.96	43.70	54.80	55.29
CRPC-FS	60.39	66.86	69.42	62.72	69.42	70.74	54.80	55.05	55.29
PCSS	68.24	69.32	69.85	69.73	70.74	71.92	56.18	56.18	60.06

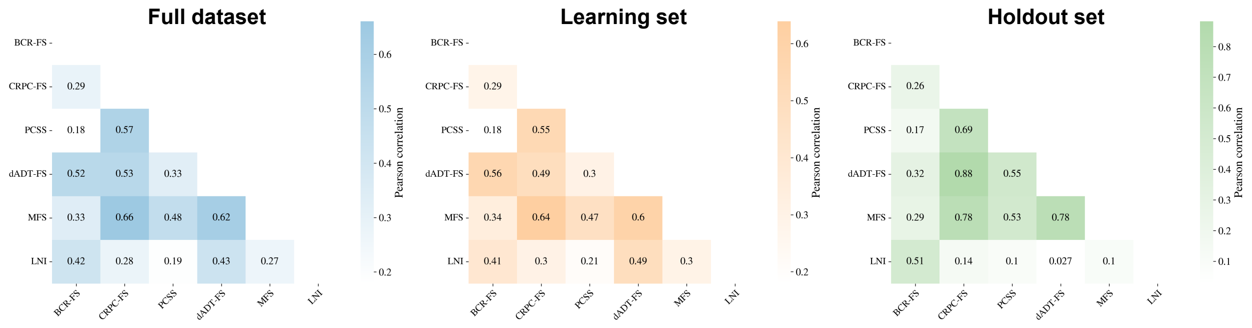

Supplementary Fig. 1 Pearson correlation between each pair of tasks for the *full dataset*, the *learning set*, and the *holdout set*. The distributions of class labels and event indicators are respectively used to compute the correlation of classification and survival tasks.

1.2.1 Biochemical recurrence-free survival (BCR-FS)

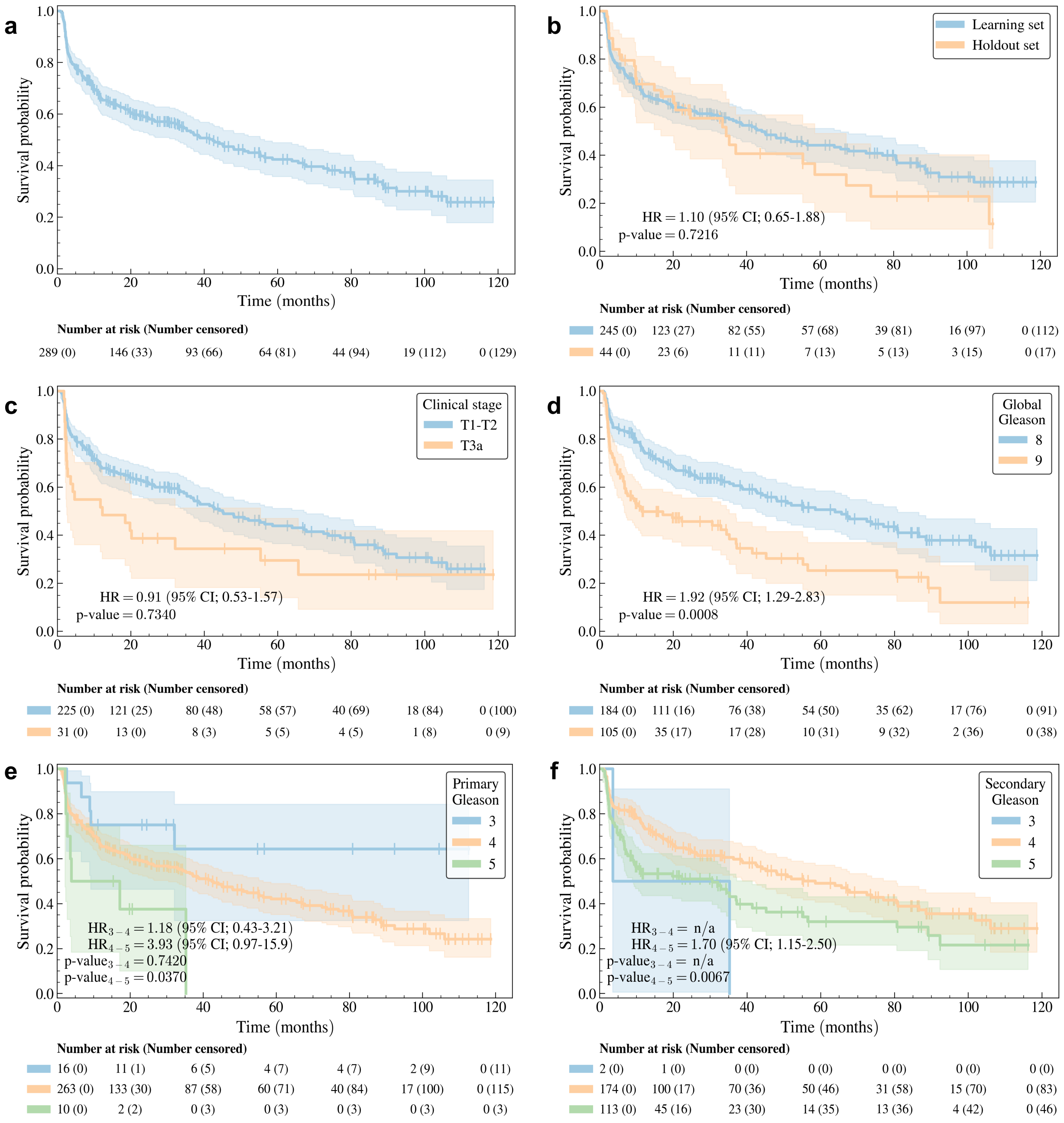

Supplementary Fig. 2 Kaplan-Meier curve [7] of the *full dataset* for the BCR-FS task using (a) no stratification and stratification based on (b) datasets, (c) clinical stage, (d) global Gleason score, (e) primary Gleason score, and (f) secondary Gleason score. The 95% confidence interval (shade) of the Kaplan-Meier curve (line) is estimated using the log hazard [8]. The *p*-value is computed using a log-rank test [9, 10], which also provides statistics to calculate the hazard ratio (HR) and its 95% confidence interval (95% CI) [11]. The `scikit-survival` [12] Python Library is used to generate the Kaplan-Meier curves and perform the tests.

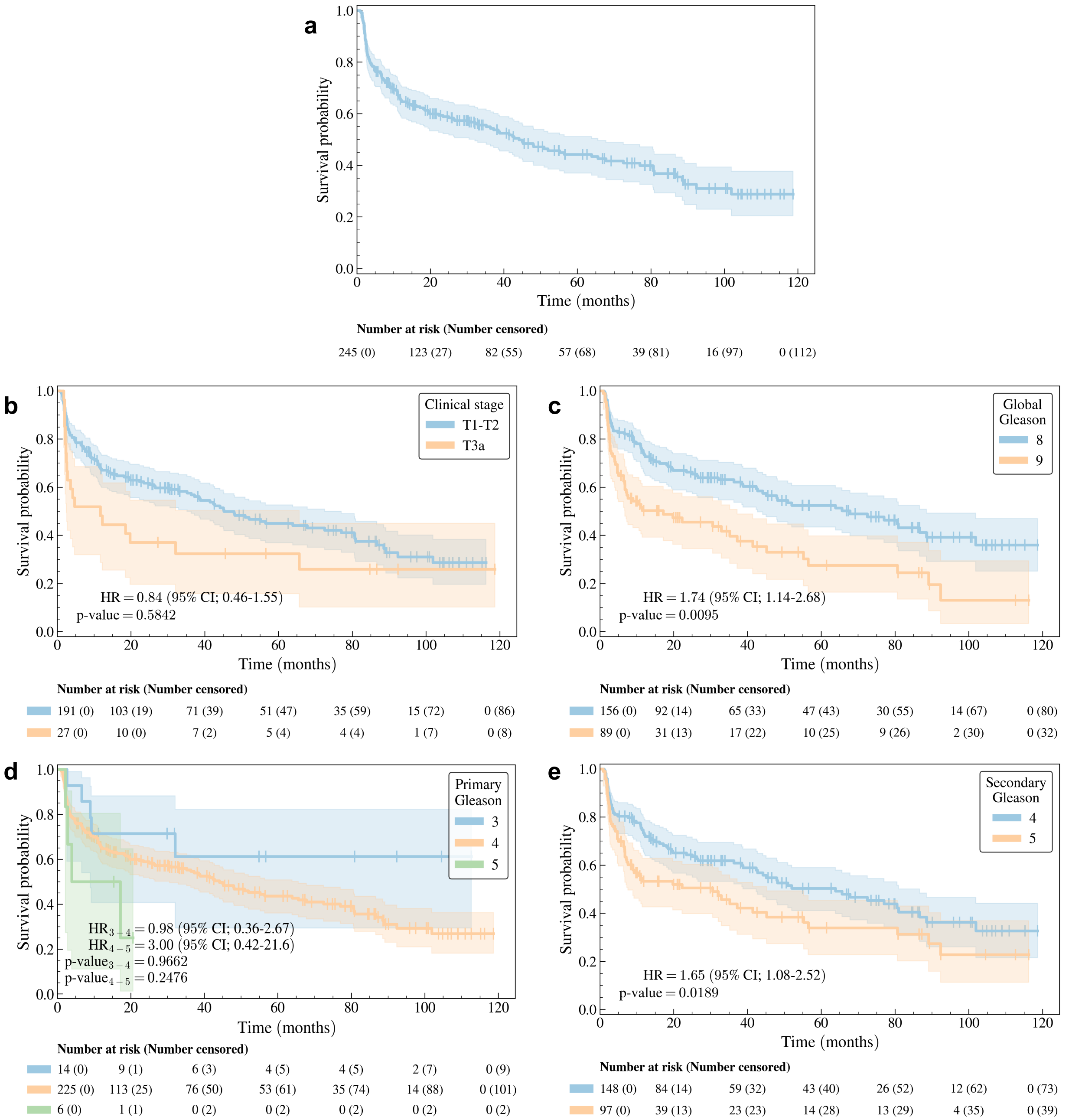

Supplementary Fig. 3 Kaplan-Meier curve [7] of the *learning set* for the BCR-FS task using (a) no stratification and stratification based on (b) clinical stage, (c) global Gleason score, (d) primary Gleason score, and (e) secondary Gleason score. The 95% confidence interval (shade) of the Kaplan-Meier curve (line) is estimated using the log hazard [8]. The *p*-value is computed using a log-rank test [9, 10], which also provides statistics to calculate the hazard ratio (HR) and its 95% confidence interval (95% CI) [11]. The `scikit-survival` [12] Python Library is used to generate the Kaplan-Meier curves and perform the tests.

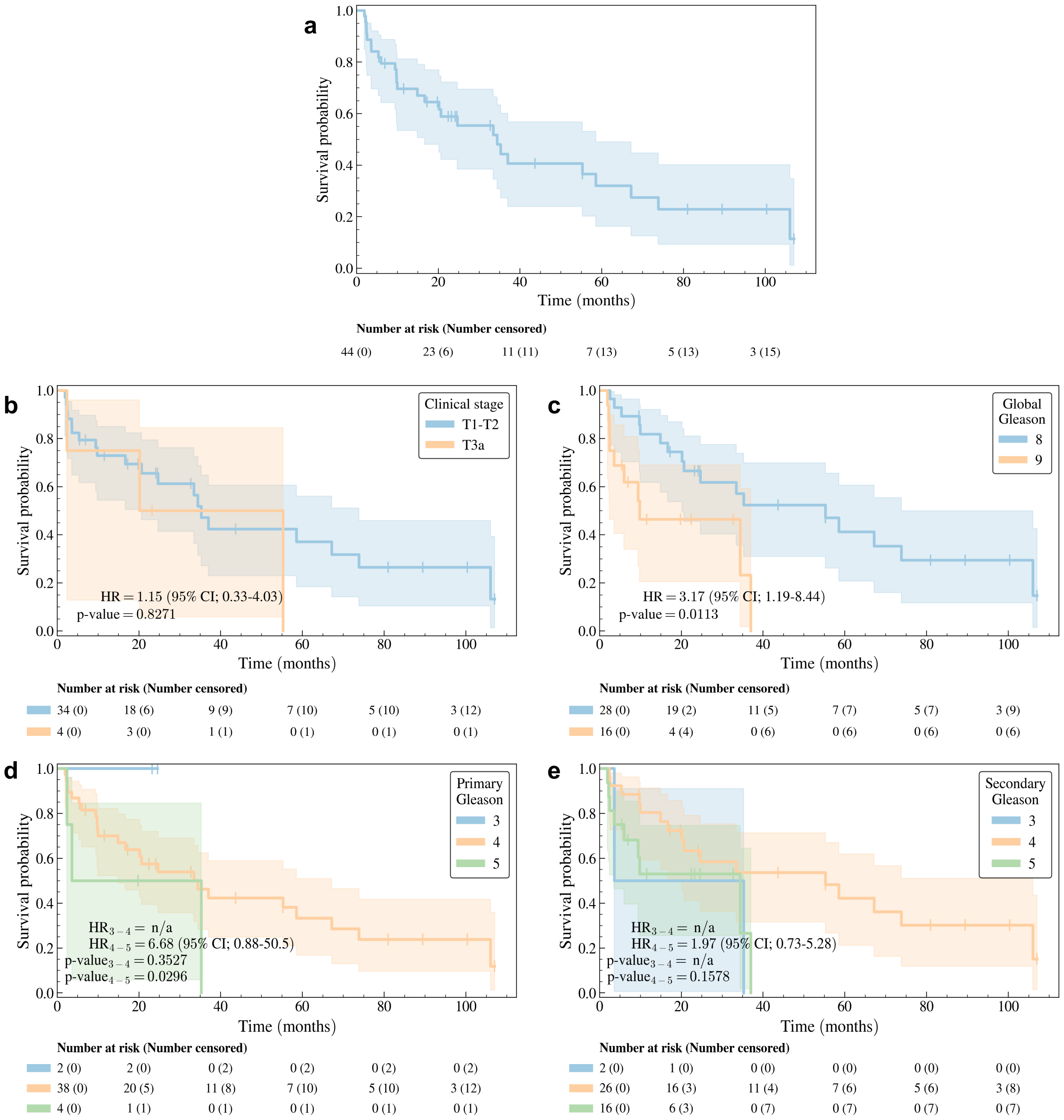

Supplementary Fig. 4 Kaplan-Meier curve [7] of the *holdout set* for the BCR-FS task using (a) no stratification and stratification based on (b) clinical stage, (c) global Gleason score, (d) primary Gleason score, and (e) secondary Gleason score. The 95% confidence interval (shade) of the Kaplan-Meier curve (line) is estimated using the log hazard [8]. The p-value is computed using a log-rank test [9, 10], which also provides statistics to calculate the hazard ratio (HR) and its 95% confidence interval (95% CI) [11]. The `scikit-survival` [12] Python Library is used to generate the Kaplan-Meier curves and perform the tests.

1.2.2 Metastasis-free survival (MFS)

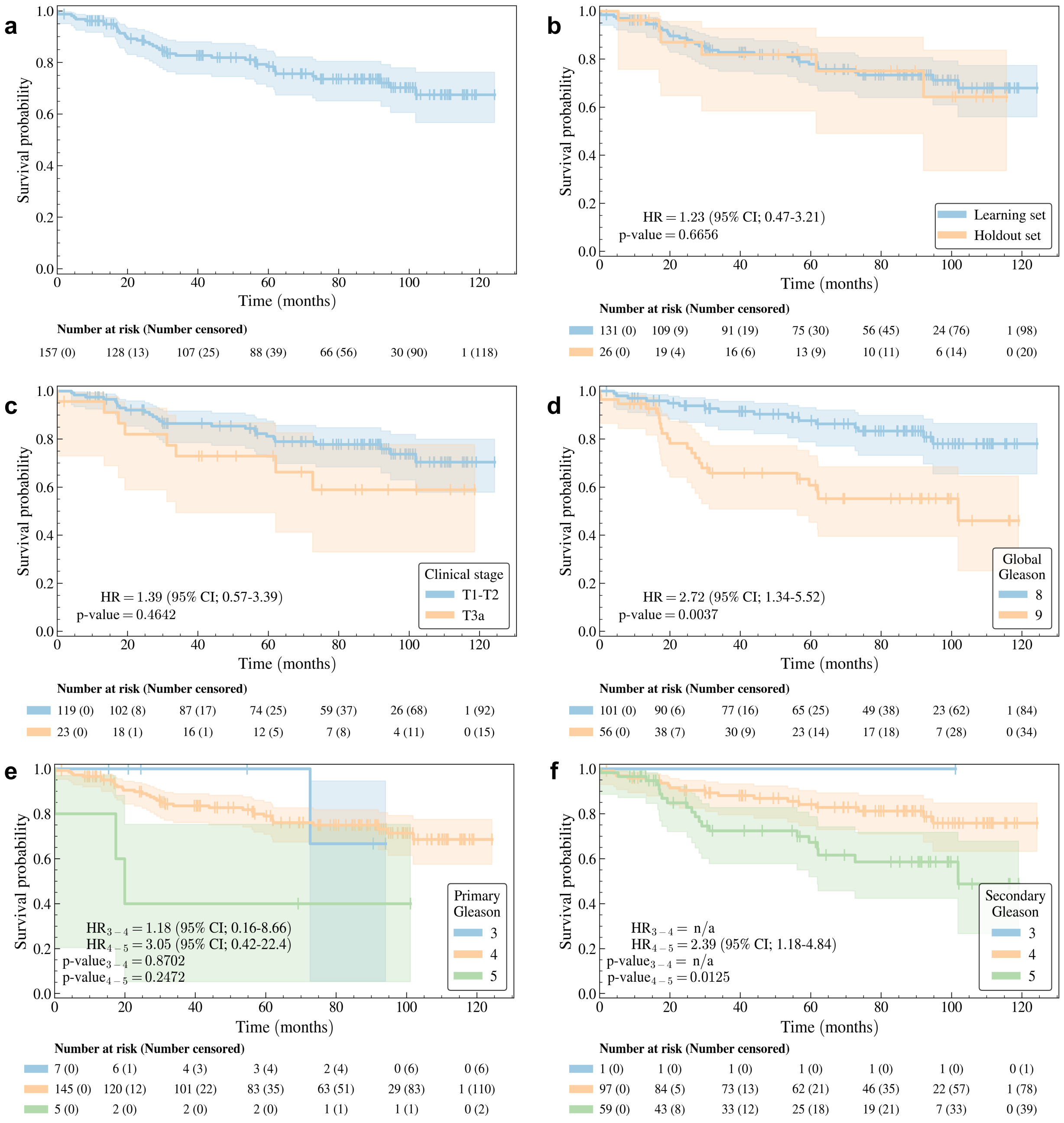

Supplementary Fig. 5 Kaplan-Meier curve [7] of the *full dataset* for the MFS task using (a) no stratification and stratification based on (b) datasets, (c) clinical stage, (d) global Gleason score, (e) primary Gleason score, and (f) secondary Gleason score. The 95% confidence interval (shade) of the Kaplan-Meier curve (line) is estimated using the log hazard [8]. The p-value is computed using a log-rank test [9, 10], which also provides statistics to calculate the hazard ratio (HR) and its 95% confidence interval (95% CI) [11]. The `scikit-survival` [12] Python Library is used to generate the Kaplan-Meier curves and perform the tests.

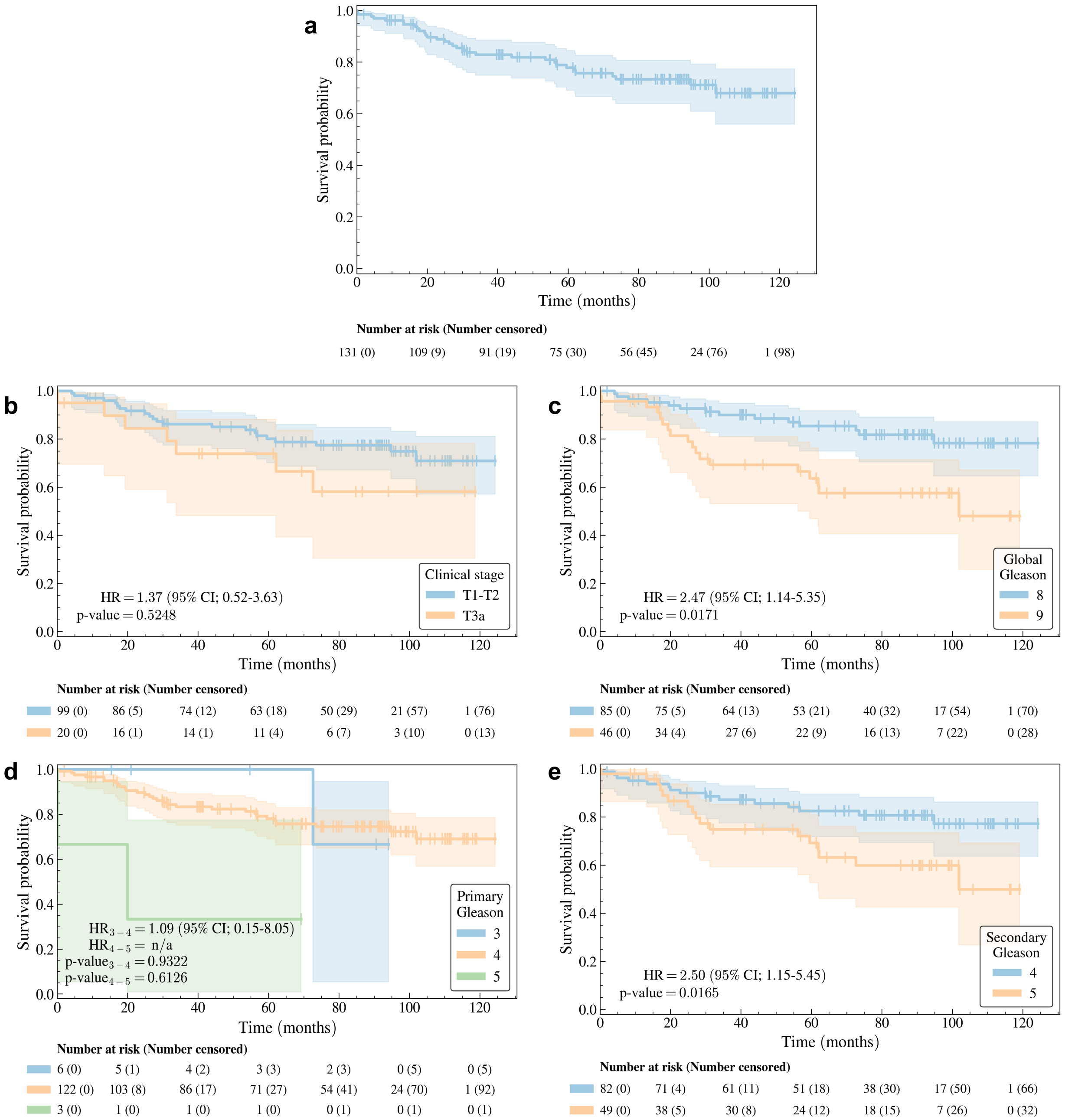

Supplementary Fig. 6 Kaplan-Meier curve [7] of the *learning set* for the MFS task using (a) no stratification and stratification based on (b) clinical stage, (c) global Gleason score, (d) primary Gleason score, and (e) secondary Gleason score. The 95% confidence interval (shade) of the Kaplan-Meier curve (line) is estimated using the log hazard [8]. The *p*-value is computed using a log-rank test [9, 10], which also provides statistics to calculate the hazard ratio (HR) and its 95% confidence interval (95% CI) [11]. The `scikit-survival` [12] Python Library is used to generate the Kaplan-Meier curves and perform the tests.

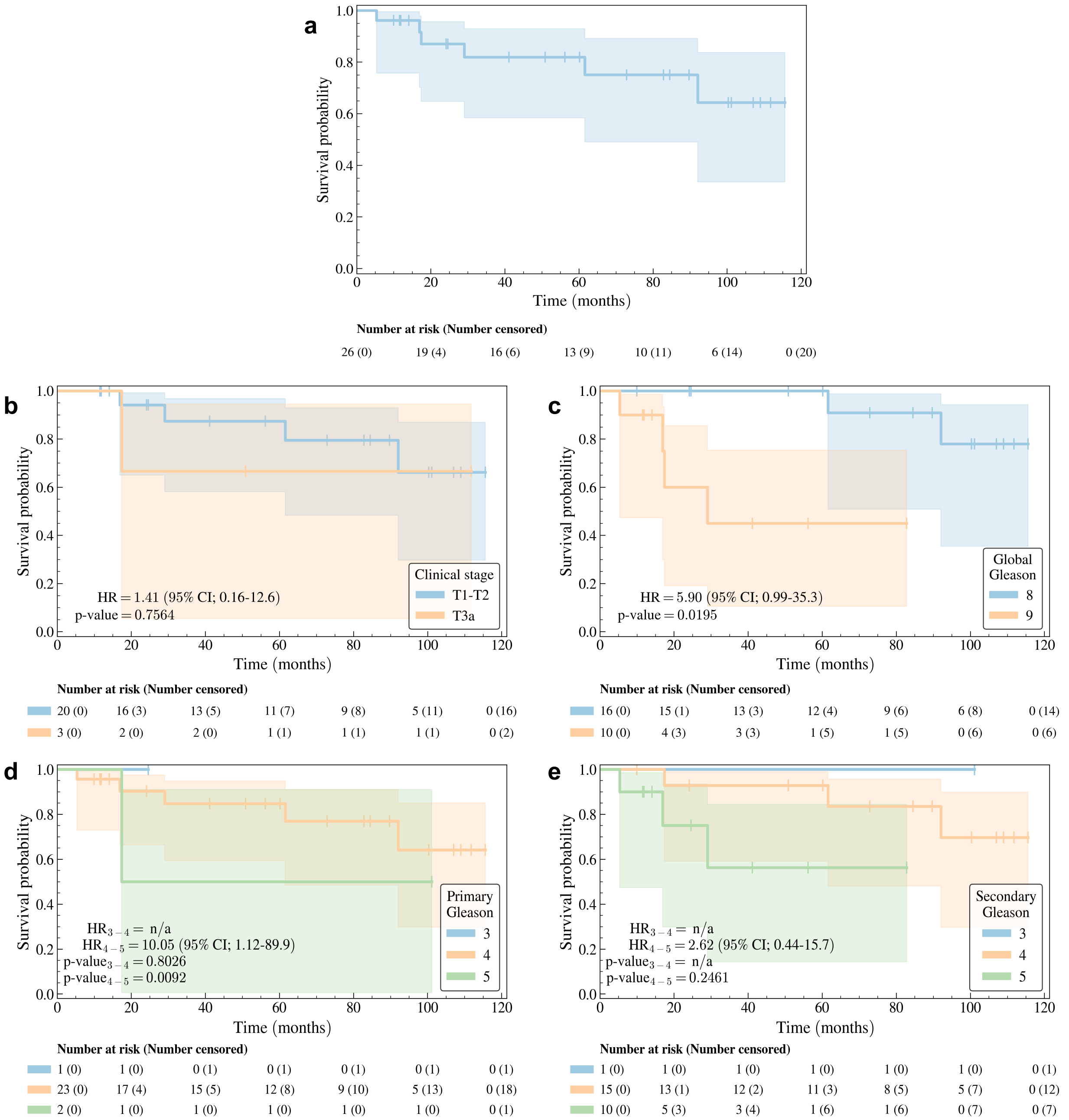

Supplementary Fig. 7 Kaplan-Meier curve [7] of the *holdout set* for the MFS task using (a) no stratification and stratification based on (b) clinical stage, (c) global Gleason score, (d) primary Gleason score, and (e) secondary Gleason score. The 95% confidence interval (shade) of the Kaplan-Meier curve (line) is estimated using the log hazard [8]. The p-value is computed using a log-rank test [9, 10], which also provides statistics to calculate the hazard ratio (HR) and its 95% confidence interval (95% CI) [11]. The `scikit-survival` [12] Python Library is used to generate the Kaplan-Meier curves and perform the tests.

1.2.3 Definitive androgen deprivation therapy-free (dADT-FS)

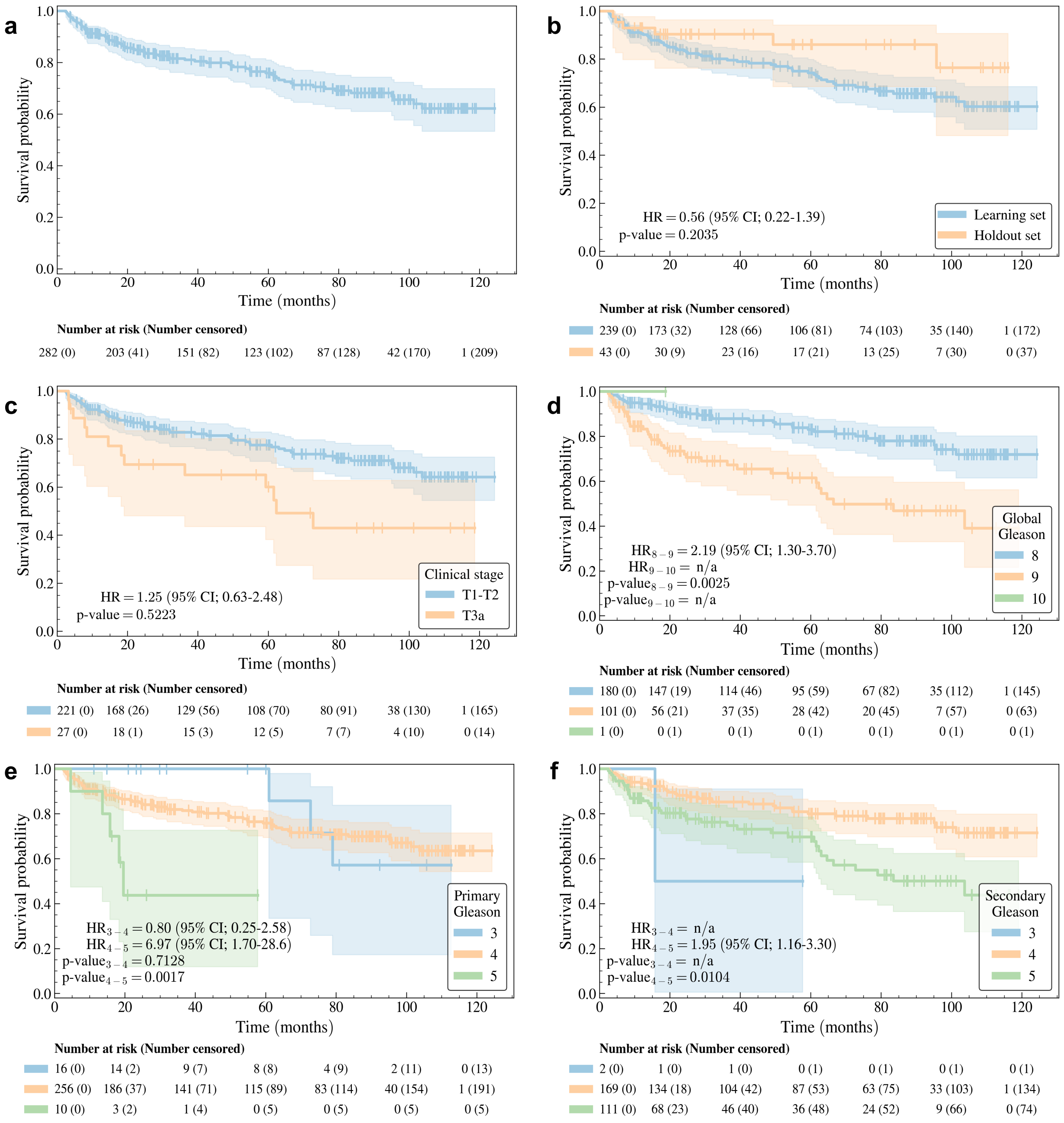

Supplementary Fig. 8 Kaplan-Meier curve [7] of the *full dataset* for the dADT-FS task using (a) no stratification and stratification based on (b) datasets, (c) clinical stage, (d) global Gleason score, (e) primary Gleason score, and (f) secondary Gleason score. The 95% confidence interval (shade) of the Kaplan-Meier curve (line) is estimated using the log hazard [8]. The *p*-value is computed using a log-rank test [9, 10], which also provides statistics to calculate the hazard ratio (HR) and its 95% confidence interval (95% CI) [11]. The `scikit-survival` [12] Python Library is used to generate the Kaplan-Meier curves and perform the tests.

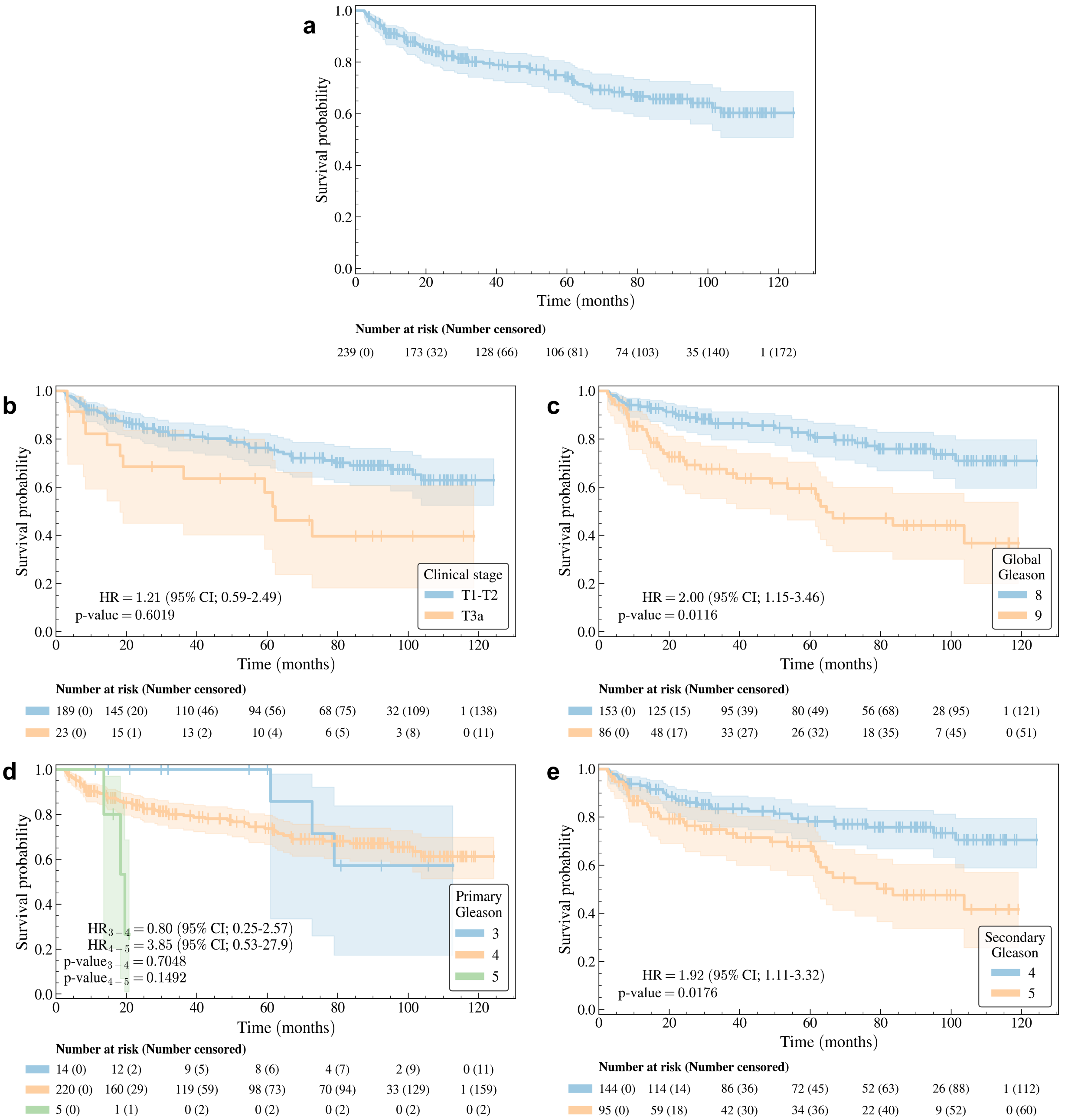

Supplementary Fig. 9 Kaplan-Meier curve [7] of the *learning set* for the dADT-FS task using (a) no stratification and stratification based on (b) clinical stage, (c) global Gleason score, (d) primary Gleason score, and (e) secondary Gleason score. The 95% confidence interval (shade) of the Kaplan-Meier curve (line) is estimated using the log hazard [8]. The p-value is computed using a log-rank test [9, 10], which also provides statistics to calculate the hazard ratio (HR) and its 95% confidence interval (95% CI) [11]. The `scikit-survival` [12] Python Library is used to generate the Kaplan-Meier curves and perform the tests.

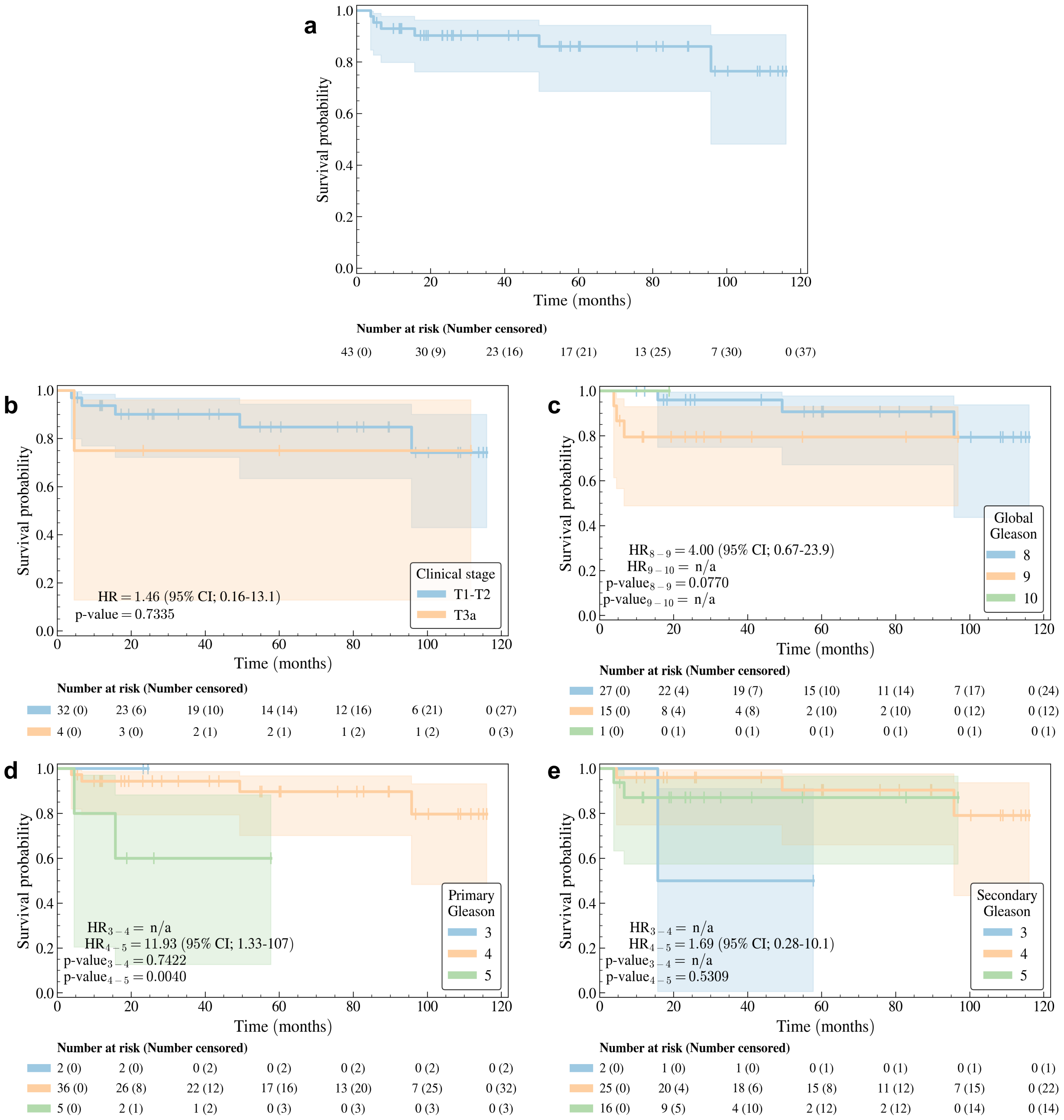

Supplementary Fig. 10 Kaplan-Meier curve [7] of the *holdout set* for the dADT-FS task using (a) no stratification and stratification based on (b) clinical stage, (c) global Gleason score, (d) primary Gleason score, and (e) secondary Gleason score. The 95% confidence interval (shade) of the Kaplan-Meier curve (line) is estimated using the log hazard [8]. The p-value is computed using a log-rank test [9, 10], which also provides statistics to calculate the hazard ratio (HR) and its 95% confidence interval (95% CI) [11]. The `scikit-survival` [12] Python Library is used to generate the Kaplan-Meier curves and perform the tests.

1.2.4 Castration-resistant prostate cancer free survival (CRPC-FS)

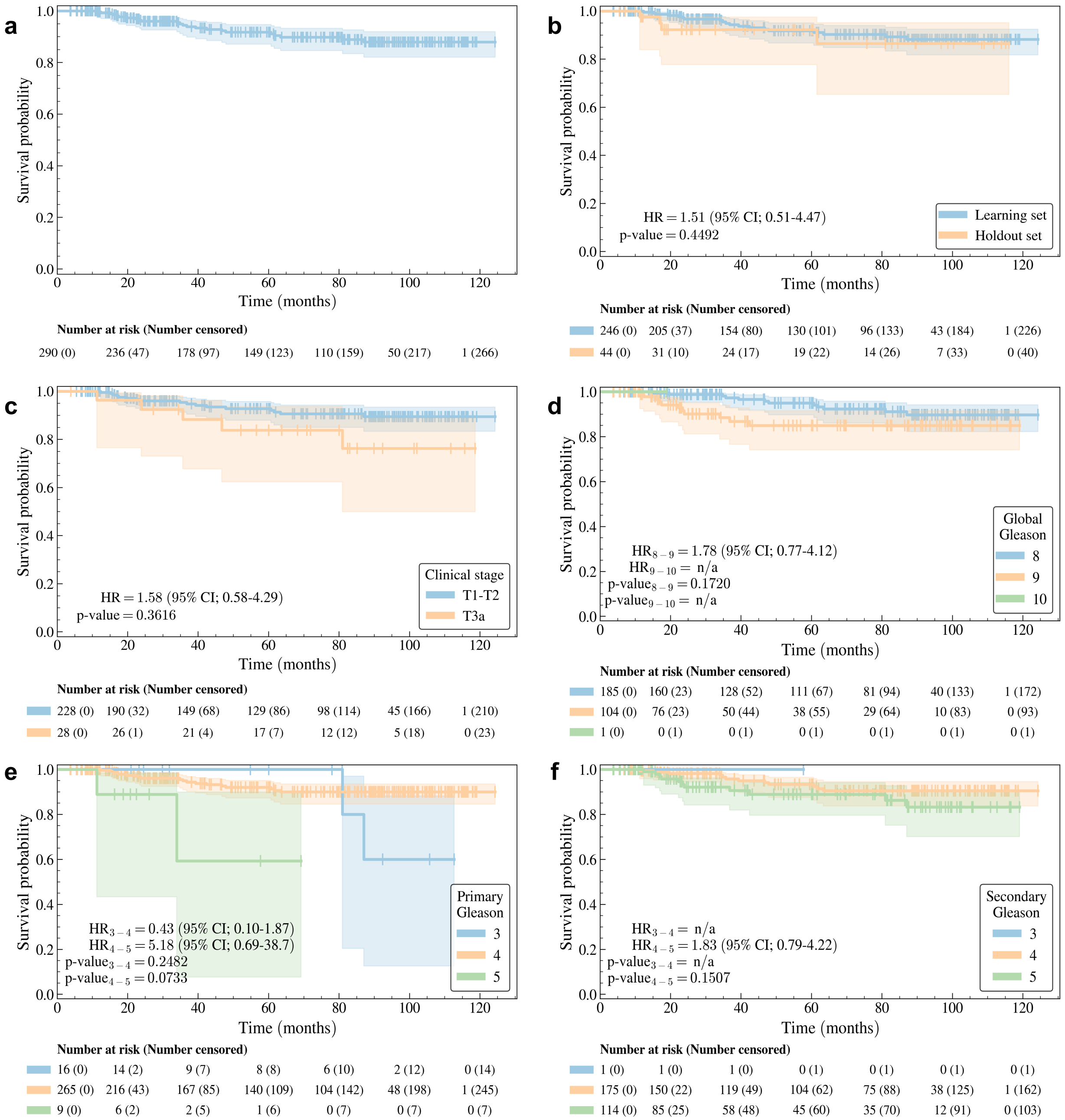

Supplementary Fig. 11 Kaplan-Meier curve [7] of the *full dataset* for the CRPC-FS task using (a) no stratification and stratification based on (b) datasets, (c) clinical stage, (d) global Gleason score, (e) primary Gleason score, and (f) secondary Gleason score. The 95% confidence interval (shade) of the Kaplan-Meier curve (line) is estimated using the log hazard [8]. The *p*-value is computed using a log-rank test [9, 10], which also provides statistics to calculate the hazard ratio (HR) and its 95% confidence interval (95% CI) [11]. The `scikit-survival` [12] Python Library is used to generate the Kaplan-Meier curves and perform the tests.

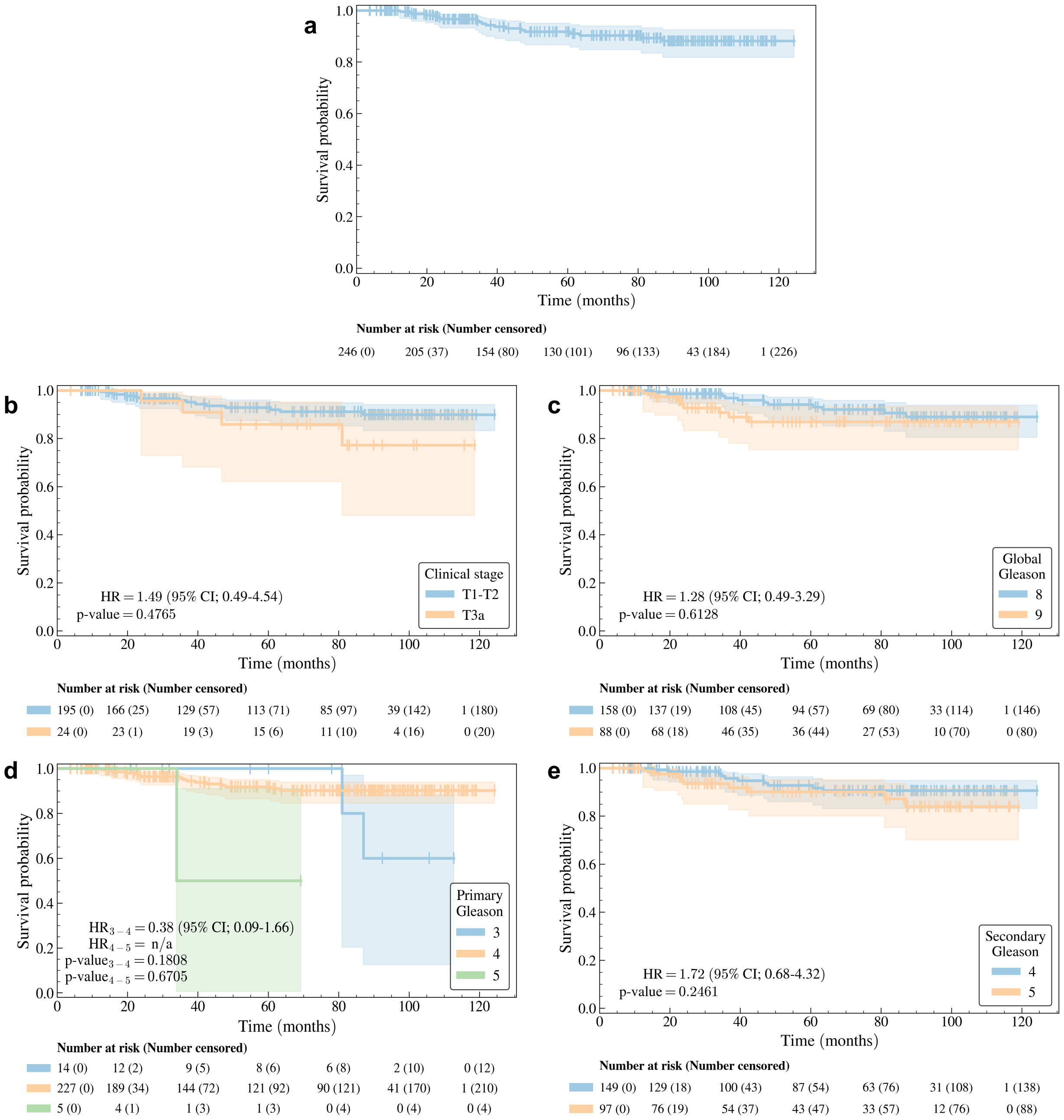

Supplementary Fig. 12 Kaplan-Meier curve [7] of the *learning set* for the CRPC-FS task using (a) no stratification and stratification based on (b) clinical stage, (c) global Gleason score, (d) primary Gleason score, and (e) secondary Gleason score. The 95% confidence interval (shade) of the Kaplan-Meier curve (line) is estimated using the log hazard [8]. The p-value is computed using a log-rank test [9, 10], which also provides statistics to calculate the hazard ratio (HR) and its 95% confidence interval (95% CI) [11]. The `scikit-survival` [12] Python Library is used to generate the Kaplan-Meier curves and perform the tests.

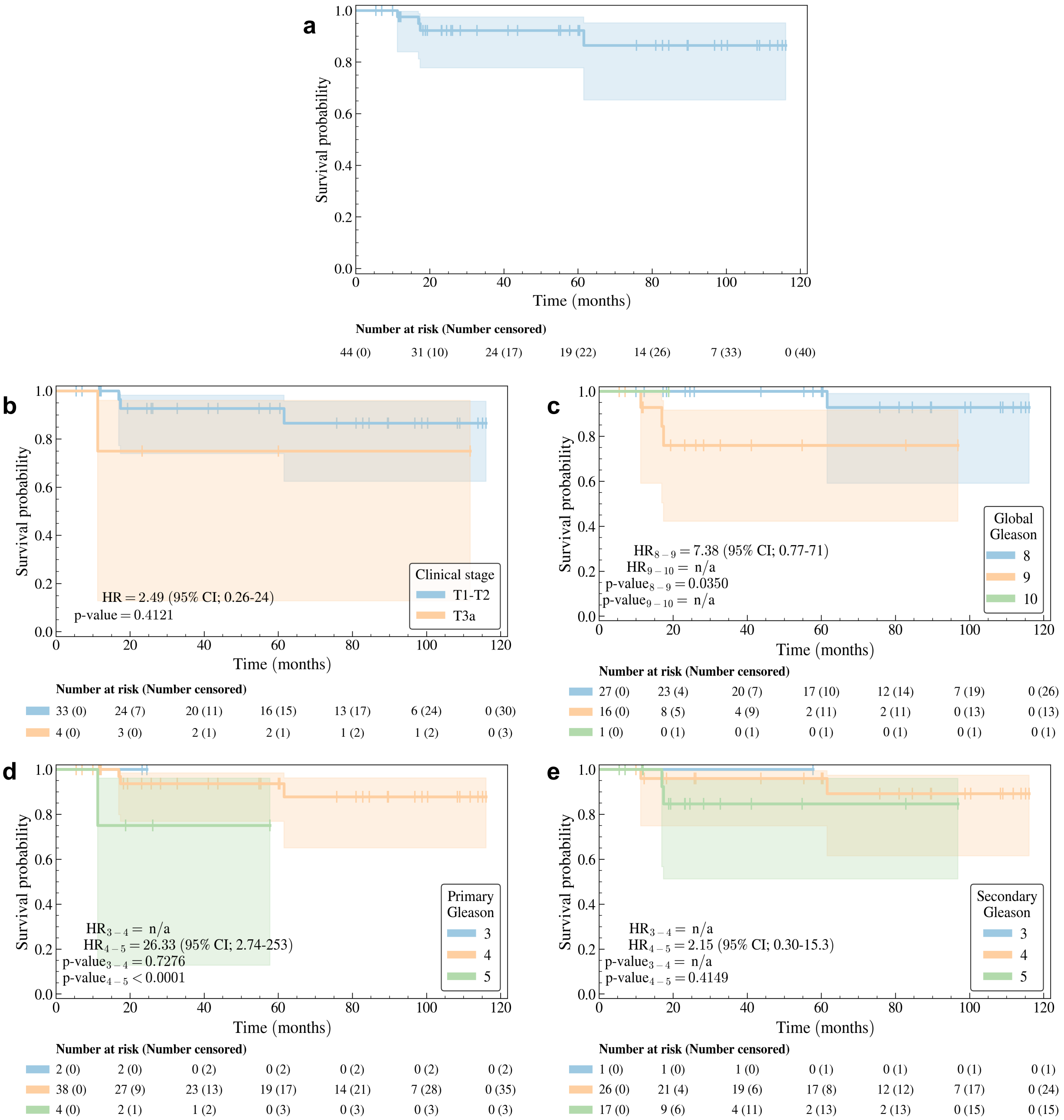

Supplementary Fig. 13 Kaplan-Meier curve [7] of the *holdout set* for the CRPC-FS task using (a) no stratification and stratification based on (b) clinical stage, (c) global Gleason score, (d) primary Gleason score, and (e) secondary Gleason score. The 95% confidence interval (shade) of the Kaplan-Meier curve (line) is estimated using the log hazard [8]. The p-value is computed using a log-rank test [9, 10], which also provides statistics to calculate the hazard ratio (HR) and its 95% confidence interval (95% CI) [11]. The `scikit-survival` [12] Python Library is used to generate the Kaplan-Meier curves and perform the tests.

1.2.5 Prostate cancer-specific survival (PCSS)

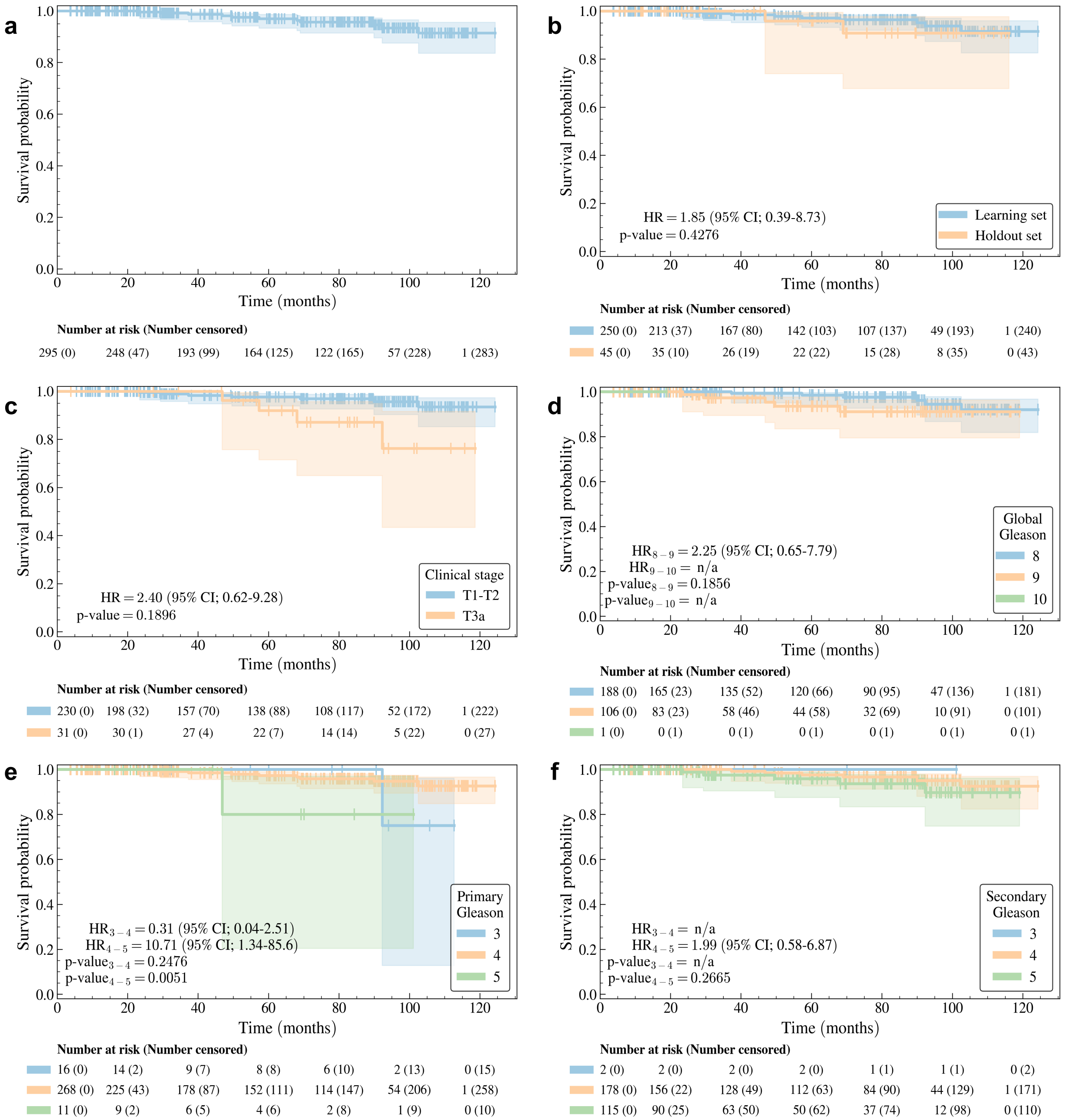

Supplementary Fig. 14 Kaplan-Meier curve [7] of the *full dataset* for the PCSS task using (a) no stratification and stratification based on (b) datasets, (c) clinical stage, (d) global Gleason score, (e) primary Gleason score, and (f) secondary Gleason score. The 95% confidence interval (shade) of the Kaplan-Meier curve (line) is estimated using the log hazard [8]. The *p*-value is computed using a log-rank test [9, 10], which also provides statistics to calculate the hazard ratio (HR) and its 95% confidence interval (95% CI) [11]. The `scikit-survival` [12] Python Library is used to generate the Kaplan-Meier curves and perform the tests.

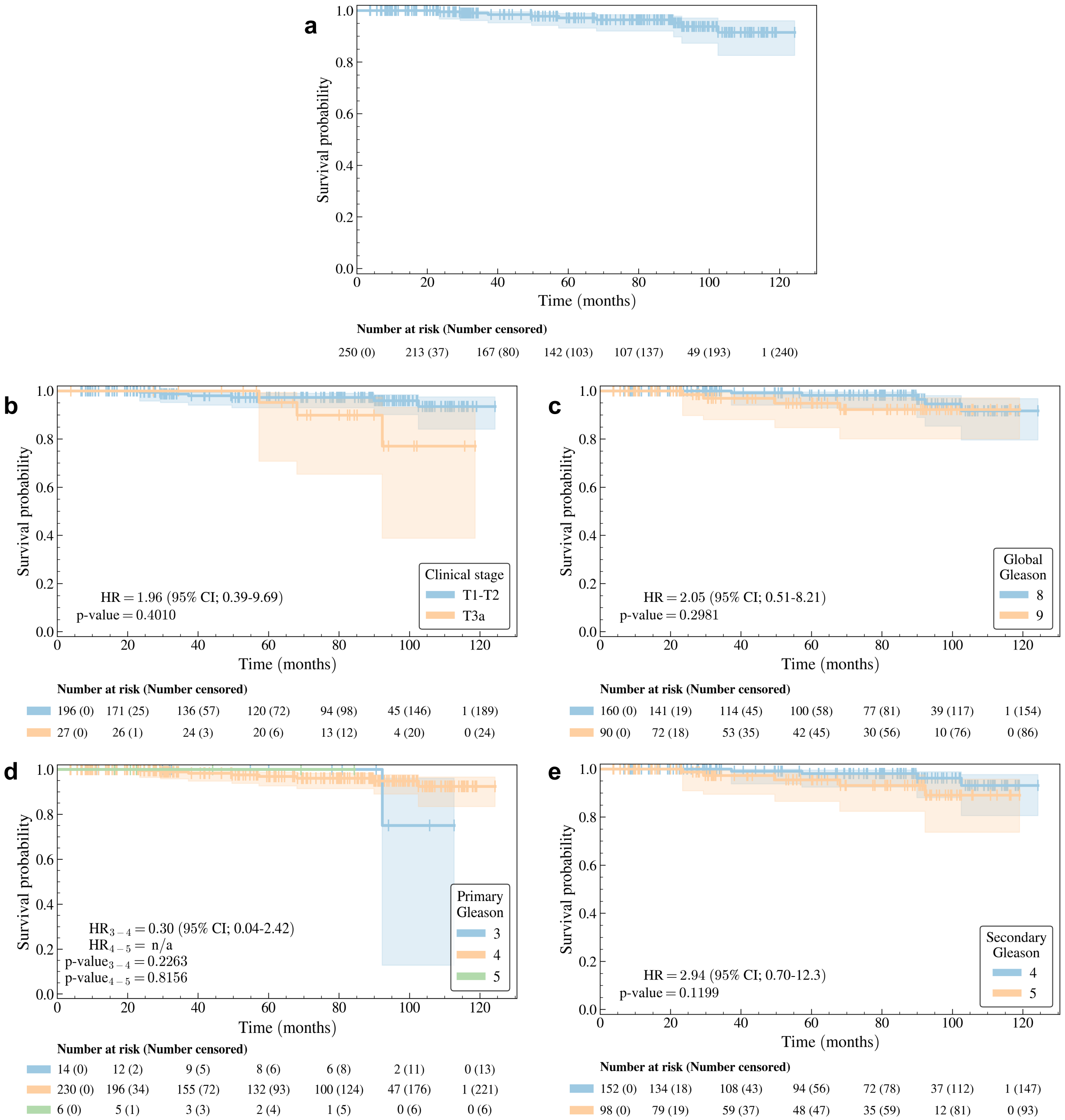

Supplementary Fig. 15 Kaplan-Meier curve [7] of the *learning set* for the PCSS task using (a) no stratification and stratification based on (b) clinical stage, (c) global Gleason score, (d) primary Gleason score, and (e) secondary Gleason score. The 95% confidence interval (shade) of the Kaplan-Meier curve (line) is estimated using the log hazard [8]. The p-value is computed using a log-rank test [9, 10], which also provides statistics to calculate the hazard ratio (HR) and its 95% confidence interval (95% CI) [11]. The `scikit-survival` [12] Python Library is used to generate the Kaplan-Meier curves and perform the tests.

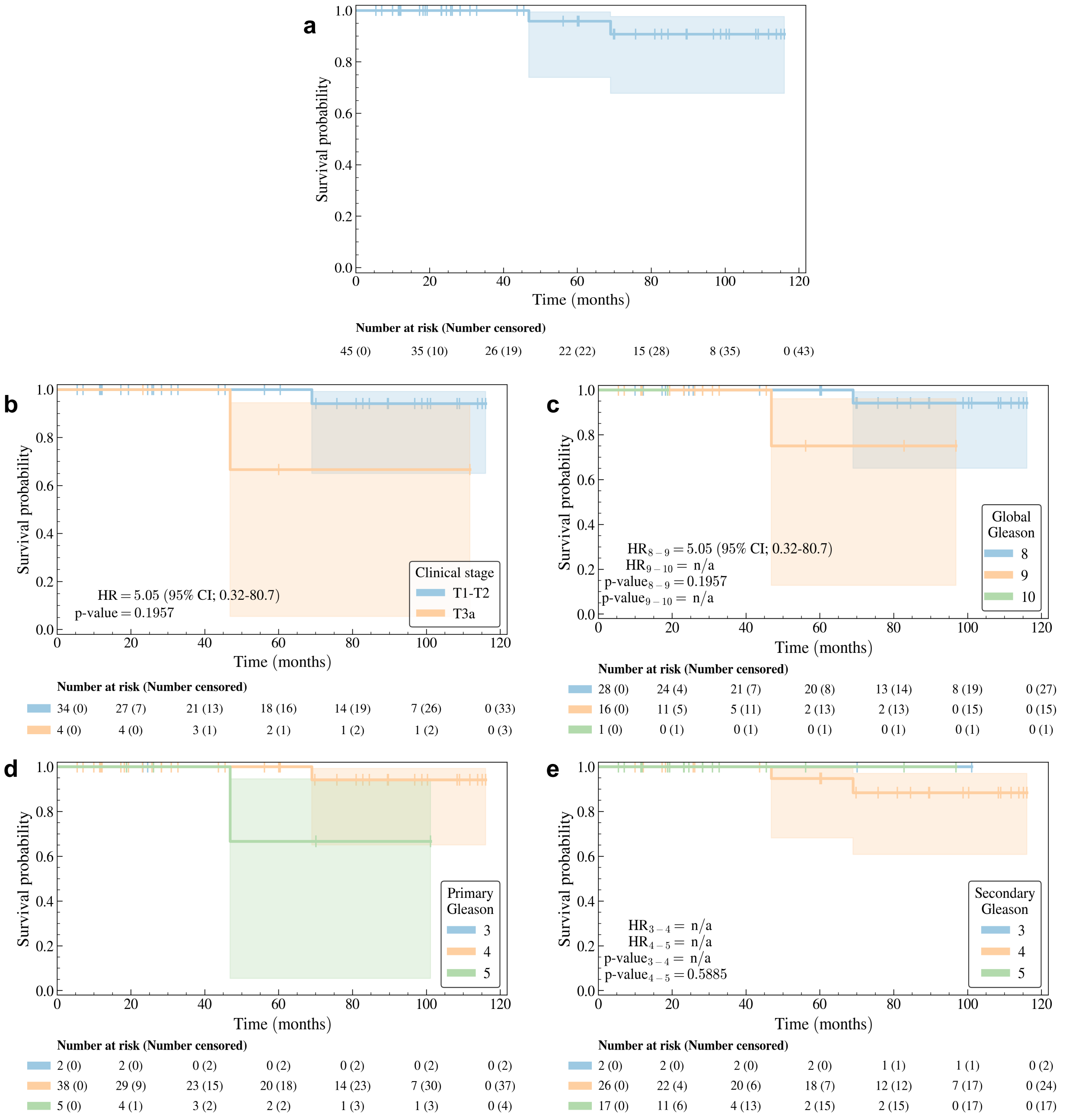

Supplementary Fig. 16 Kaplan-Meier curve [7] of the *holdout set* for the PCSS task using (a) no stratification and stratification based on (b) clinical stage, (c) global Gleason score, (d) primary Gleason score, and (e) secondary Gleason score. The 95% confidence interval (shade) of the Kaplan-Meier curve (line) is estimated using the log hazard [8]. The p-value is computed using a log-rank test [9, 10], which also provides statistics to calculate the hazard ratio (HR) and its 95% confidence interval (95% CI) [11]. The `scikit-survival` [12] Python Library is used to generate the Kaplan-Meier curves and perform the tests.

1.3 Visual comparison between the *learning set* and the *holdout set*

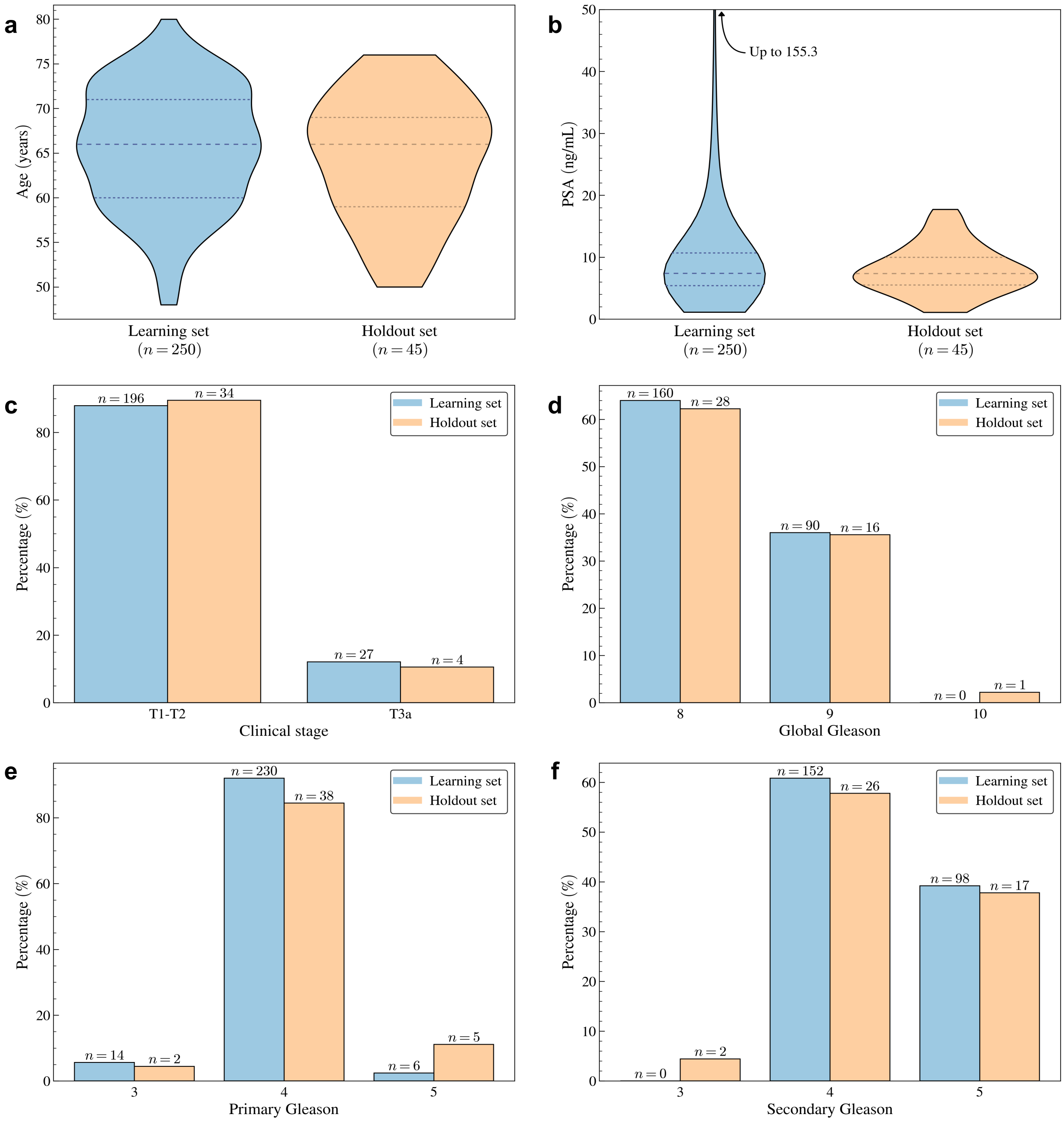

Supplementary Fig. 17 Comparison of the distribution of (a) age, (b) prostate-specific antigen (PSA), (c) clinical stage, (d) global Gleason score, (e) primary Gleason score, and (f) secondary Gleason score between the *learning set* and the *holdout set*. Note that there is 1 patient with a missing PSA and 34 patients with missing clinical stage in the *full dataset*.

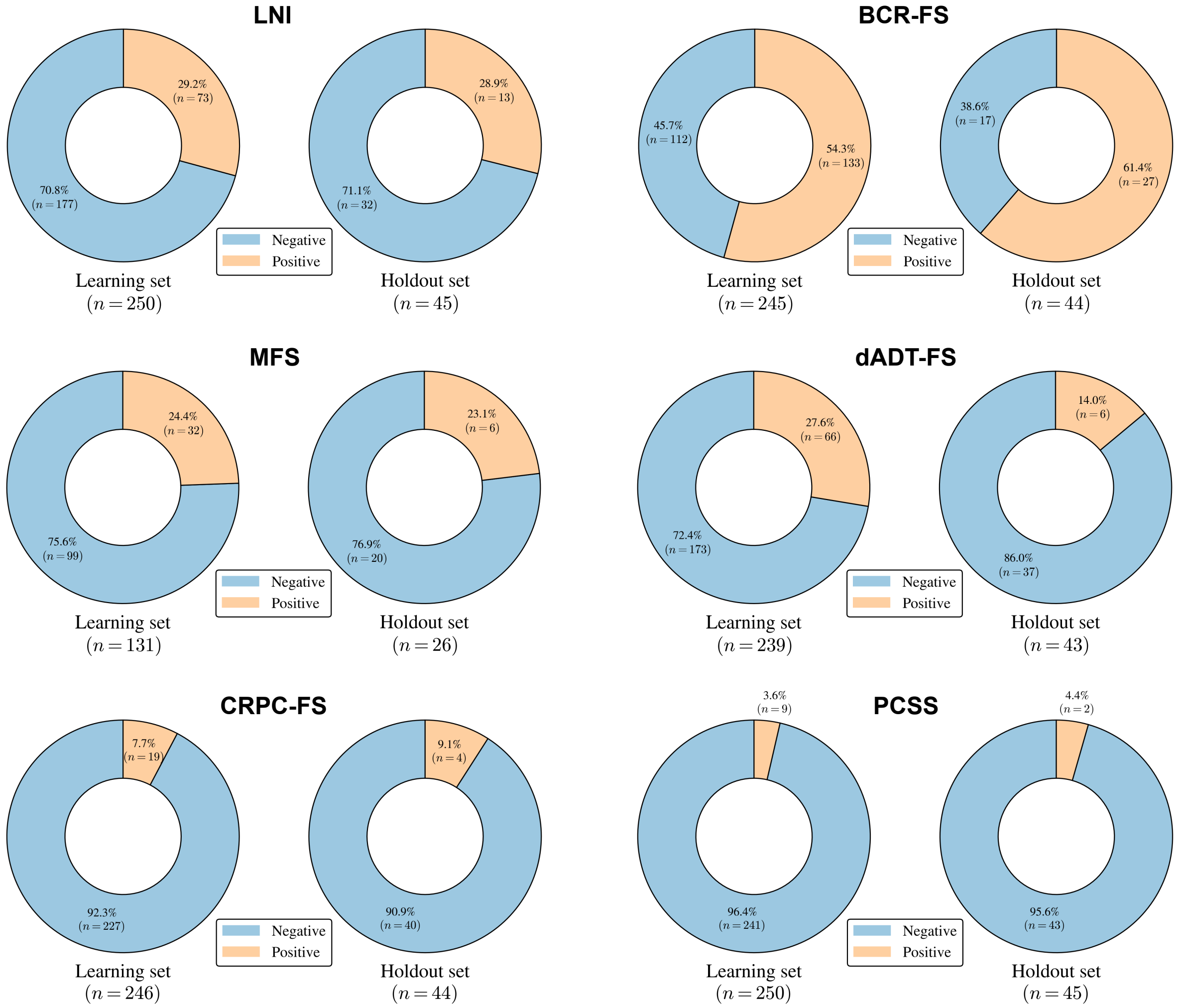

Supplementary Fig. 18 Comparison of the distribution of class labels and event indicators between the *learning set* and the *holdout set* for the classification and survival tasks, respectively.

1.4 Statistical analysis between the *learning set* and the *holdout set*

Supplementary Table 9 Statistical analysis comparing the distribution of each clinical feature between the *learning set* and the *holdout set* using the Mann-Whitney U test [1] and the χ^2 test [2].

Feature	Test	p-value
Age	Mann–Whitney U	4×10^{-1}
PSA	Mann–Whitney U	6×10^{-1}
Clinical stage	χ^2	1×10^{0}
Global Gleason	χ^2	6×10^{-2}
Primary Gleason	χ^2	2×10^{-2}
Secondary Gleason	χ^2	4×10^{-3}

Supplementary Table 10 Statistical analysis comparing the distribution of class labels and event indicators between the *learning set* and the *holdout set* using the χ^2 test [2] and the log-rank test [9, 10] for classification and survival tasks, respectively.

Task	Test	p-value
LNI	χ^2	1×10^{0}
BCR-FS	Log-rank	7×10^{-1}
MFS	Log-rank	7×10^{-1}
dADT-FS	Log-rank	2×10^{-1}
CRPC-FS	Log-rank	4×10^{-1}
PCSS	Log-rank	4×10^{-1}

2 Model hyperparameters

2.1 Hyperparameter search space

Supplementary Table 11 Fixed hyperparameters common to all models.

Hyperparameter	Value
Activation	PReLU
Batch size	16
Epochs	100
Learning rate scheduler	Exponential
Normalization	Instance
Optimizer	Adam
Patience	20
Regularization	L2

Supplementary Table 12 Search space for hyperparameters of both standalone MLPs and those integrated within the SN.

Hyperparameter	Type	Search space
Dropout	Float	$[0.05, 0.25]$
Learning rate	Float[1]	$[0.0001, 0.01]$
Layers	Integer	$\{0, 1, 2, 3\}$
Neurons	Integer	$\{5, 10, 15, 20\}$
Weight decay	Float[1]	$[0.0001, 0.01]$
α	Float[1]	$[0.00001, 0.01]$
γ	Fixed	0.99

[1]The value is sampled from the range in the log domain.

Supplementary Table 13 Search space for hyperparameters of the Bayesian MLPs integrated in the BSN.

Hyperparameter	Type	Search space
Dropout	Float	$[0, 0.25]$
Learning rate	Float[1]	$[0.0001, 0.01]$
Layers	Integer	$\{0, 1, 2, 3\}$
Neurons	Integer	$\{5, 10, 15, 20\}$
Weight decay	Float[1]	$[0.0001, 0.01]$
α	Float[1]	$[0.00001, 0.01]$
γ	Fixed	0.99
Temperature	Float[1]	$[0.0001, 0.1]$

[1]The value is sampled from the range in the log domain.

Supplementary Table 14 Search space for hyperparameters of the U-Net.

Hyperparameter	Type	Search space
Channels	Fixed	(64, 128, 256, 512, 1024)
Dropout	Float	$[0, 0.3]$
Kernel size	Fixed	3
Learning rate	Float[1]	[0.0005, 0.005]
Residual units	Fixed	3
Weight decay	Fixed	0.01
α	Fixed	0
γ	Fixed	0.99

[1]The value is sampled from the range in the log domain.

Supplementary Table 15 Search space for hyperparameters of the Bayesian U-Net.

Hyperparameter	Type	Search space
Channels	Fixed	(64, 128, 256, 512, 1024)
Dropout	Float	$[0, 0.1]$
Kernel size	Fixed	3
Learning rate	Float[1]	[0.0005, 0.005]
Residual units	Fixed	3
Temperature	Fixed	0.0001
Weight decay	Fixed	0.01
α	Fixed	0
γ	Fixed	0.99

[1]The value is sampled from the range in the log domain.

Supplementary Table 16 Search space for hyperparameters of the U-NEXtractor.

Hyperparameter	Type	Search space
Channels	Fixed	(64, 128, 256, 512, 1024)
Dropout (CNN)	Float	$[0.2, 0.8]$
Dropout (FNN)	Float	$[0.1, 0.4]$
Kernel size	Fixed	3
Learning rate	Float[1]	$[0.0001, 0.001]$
Loss weights ($\omega_{\text{prognosis}}, \omega_{\text{segmentation}}$)	Categorical	$\{(0.25, 0.75), (0.33, 0.67), (0.5, 0.5), (0.67, 0.33), (0.75, 0.25)\}$
Residual units	Fixed	2
Weight decay	Float[1]	$[0.001, 0.1]$
α	Float[1]	$[0.0001, 0.01]$
γ	Fixed	0.95

[1]The value is sampled from the range in the log domain.

Supplementary Table 17 Search space for hyperparameters of the Bayesian U-NEXtractor.

Hyperparameter	Type	Search space
Channels	Fixed	(64, 128, 256, 512, 1024)
Dropout (CNN)	Float	$[0, 0.6]$
Dropout (FNN)	Float	$[0, 0.3]$
Kernel size	Fixed	3
Learning rate	Float[1]	$[0.0001, 0.001]$
Loss weights ($\omega_{\text{prognosis}}, \omega_{\text{segmentation}}$)	Categorical	$\{(0.25, 0.75), (0.33, 0.67), (0.5, 0.5), (0.67, 0.33), (0.75, 0.25)\}$
Residual units	Fixed	2
Temperature (Prognosis task)	Float[1]	$[0.0001, 0.1]$
Temperature (Segmentation task)	Fixed	0.0001
Weight decay	Float[1]	$[0.001, 0.1]$
α	Float[1]	$[0.0001, 0.01]$
γ	Fixed	0.95

[1]The value is sampled from the range in the log domain.

2.2 Selected hyperparameter values

Supplementary Table 18 Hyperparameters of the Bayesian MLPs integrated in the BSN trained with the *learning set*.

Hyperparameter	LNI	BCR-FS	MFS	dADT-FS	CRPC-FS	PCSS
Dropout	0.10	0.10	0.05	0.15	0.05	0.05
Learning rate	0.002	0.002	0.001	0.002	0.002	0.002
Layers	2	1	0	2	1	2
Neurons	10	15	0	10	10	10
Weight decay	0.001	0.001	0.001	0.001	0.005	0.001
α	0.001	0.001	0.001	0.005	0.0005	0.001
γ	0.99	0.99	0.99	0.99	0.99	0.99
Temperature	0.0001	0.001	0.0001	0.0001	0.001	0.001

Supplementary Table 19 Hyperparameters of the Bayesian U-NEXtractor integrated in the BSN trained with the *learning set*.

Hyperparameter	Value
Channels	(64, 128, 256, 512, 1024)
Dropout (CNN)	0.4
Dropout (FNN)	0.1
Kernel size	3
Learning rate	0.0001
Loss weights ($\omega_{\text{prognosis}}, \omega_{\text{segmentation}}$)	$(0.5, 0.5)$
Residual units	2
Temperature (BCR-FS task)	0.01
Temperature (Segmentation task)	0.0001
Weight decay	0.01
α	$[0.0001, 0.01]$
γ	0.95

Supplementary Table 20 Hyperparameters of the Bayesian U-Net integrated in the BSN trained with the *learning set*.

Hyperparameter	Value
Channels	(64, 128, 256, 512, 1024)
Dropout	0
Kernel size	3
Learning rate	0.001
Residual units	3
Temperature	0.0001
Weight decay	0.01
α	0
γ	0.99

3 Handcrafted radiomics extraction parameters

Supplementary Table 21 Parameters for the extraction of handcrafted radiomic features on the CT image using the `pyradiomics` [13] Python library. Description of the parameters is available on the web page of the `pyradiomics` package at https://pyradiomics.readthedocs.io/en/latest/features.html.

Parameter	Value
Bin width	25
Interpolator	B-spline
Sigma	$[1, 2, 3, 4, 5]$
Resegment range	$[-500, \infty]$
Image type	Original
Feature class	Shape, first-order, glcm, glrlm, glszm, gldm, ngtdm

Supplementary Table 22 Parameters for the extraction of handcrafted radiomic features on the PET image using the `pyradiomics` [13] Python library. Description of the parameters is available on the web page of the `pyradiomics` package at https://pyradiomics.readthedocs.io/en/latest/features.html.

Parameter	Value
Bin width	1
Interpolator	B-spline
Sigma	$[1, 2, 3, 4, 5]$
Resegment range	$[0, 25]$
Image type	Original
Feature class	Shape, first-order, glrlm, glszm, gldm, ngtdm

4 Experiments results

4.1 Selected handcrafted radiomic features

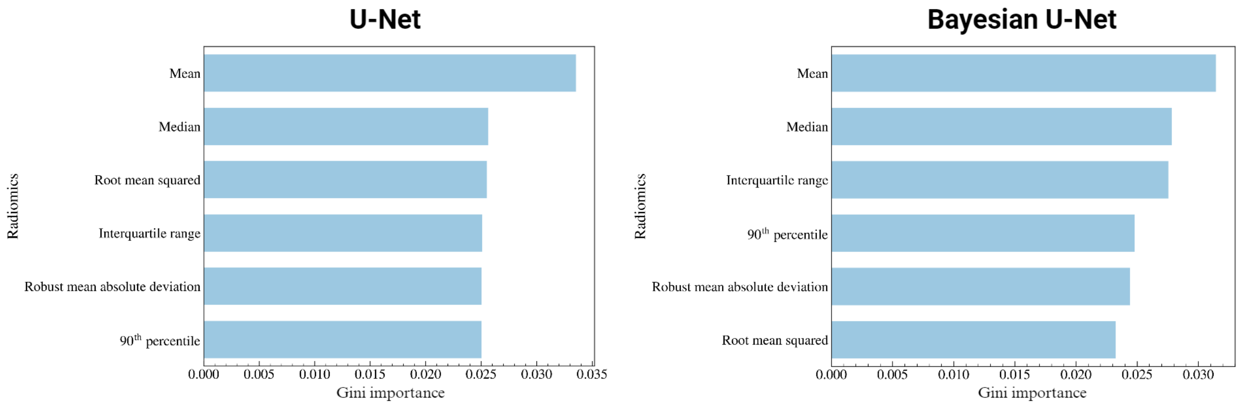

Supplementary Fig. 19 Gini importance [14] of the 6 most important handcrafted radiomic features extracted from the PET image in the region defined by the prostate segmentation map generated by the U-Net and the Bayesian U-Net. The Gini importance is obtained from a random forest classifier with 10,000 trees implemented with the `scikit-learn` [15] Python library and trained to predict LNI using 200 radiomic features extracted on both CT and PET images. The 6 most important features all come from the PET image. Both the segmentation networks and the random forest classifier are trained using the *learning set*.

4.2 Model performance

Supplementary Table 23 Performance of the models for the LNI task. See Table 1 for the full caption.

		Model	Data	LNI			
				AUC	BA	TPR	TNR
Test sets	(A)	MSKCC	CD	70±7	65±5	67±17	62±21
		CAPRA	CD	62±4	52±5	30±21	74±28
	(B)	MLP	CD	69±7	64±6	66±8	62±14
		MLP	CD+HCR	**72±5**	**67±3**	60±11	74±6
		MLP	CD+DLR	54±7	52±5	61±25	42±27
	(C)	SN	(B)'s best[1]	**72±5**	**67±3**	60±11	74±6
		BSN	(B)'s best[1]	71±4	67±4	58±14	76±10
	(D)	$BSN_{t=12}$	(B)'s best[1]	71±4	67±4	58±14	76±10
		$BSN_{t=24}$	(B)'s best[1]	71±4	67±4	58±14	76±10
		$BSN_{t=60}$	(B)'s best[1]	71±4	67±4	58±14	76±10
Holdout set	(E)	MSKCC	CD	60.6	52.2	23.1	81.3
		CAPRA	CD	57.7	50.0	0.0	100.0
	(F)	SN	(B)'s best[1]	65.4	65.1	61.5	68.8
		BSN	(B)'s best[1]	**66.3**	**66.7**	61.5	71.9
	(G)	$BSN_{t=12}$	(B)'s best[1]	66.3	66.7	61.5	71.9
		$BSN_{t=24}$	(B)'s best[1]	66.3	66.7	61.5	71.9
		$BSN_{t=60}$	(B)'s best[1]	66.3	66.7	61.5	71.9

[1] Section (B)'s best data is CD+HCR for LNI, CD+DLR for BCR-FS and CD for MFS, dADT-FS, CRPC-FS and PCSS.

Supplementary Table 24 Results of intermediate experiments on the *test sets* showing pairwise comparison of the performance of different models. The prostate segmentation maps generated by the U-Net, which are used to compute automatic handcrafted radiomic features (AHCR), achieved an average Dice similarity coefficient (DSC) [16] of 0.842± 0.004 compared to manual contours, which are used to compute the manual handcrafted radiomic features (MHCR).

	Model	Data	Task																			
			LNI				BCR-FS			MFS			dADT-FS			CRPC-FS			PCSS			
			AUC	BA	TPR	TNR	CI	CICW	CDA	CI	CICW	CDA	CI	CICW	CDA	CI	CICW	CDA	CI	CICW	CDA
A	LR	CD	68±7	64±6	67±8	62±14	62±7	63±5	68±8	66±4	63±9	70±9	68±5	67±7	70±8	65±13	56±14	72±13	64±11	59±21	61±13
A	MLP	CD	69±7	64±6	66±8	62±14	63±7	63±5	66±6	67±6	64±12	74±8	68±6	67±5	68±8	71±10	69±7	72±9	70±16	73±23	58±34
B	MLP	MHCR	61±6	57±2	53±11	62±11	50±5	52±5	48±10	40±14	44±15	40±19	55±9	54±11	53±11	56±13	57±15	55±14	55±25	63±27	51±26
B	MLP	AHCR	58±10	57±7	31±22	82±10	48±5	49±4	45±2	47±9	43±10	51±14	54±5	53±5	56±6	61±18	60±18	62±19	49±28	50±30	51±30
C	MLP	CD+MHCR	73±4	67±4	63±11	72±11	59±9	59±8	62±9	57±7	54±6	58±9	61±10	61±8	62±7	68±9	70±9	67±8	68±9	70±9	67±8
C	MLP	CD+AHCR	72±5	67±3	60±11	74±6	58±7	58±4	58±9	58±4	53±9	62±6	64±12	62±10	66±11	67±10	65±10	69±17	70±10	64±20	74±15
D	CNN	CT+PET	55±5	52±2	50±43	54±45	53±4	52±5	58±4	53±9	54±9	53±16	54±8	54±9	54±10	45±20	50±17	42±23	50±26	45±24	52±33
D	U-NEX	CT+PET	50±3	50±2	31±37	69±39	55±3	56±3	60±11	61±12	63±13	61±17	50±4	53±45	54±9	49±10	54±14	46±11	32±13	44±20	30±20

Supplementary Table 25 Dice similarity coefficient (DSC) [16] on the *training set*, the *validation set*, and the *holdout set* of segmentation models trained with the *learning set*.

	Training set			Validation set			Holdout set		
Model	Mean (Median)	Min-Max	Std	Mean (Median)	Min-Max	Std	Mean (Median)	Min-Max	Std
U-Net	0.947 (0.954)	0.778 - 0.972	0.025	0.843 (0.849)	0.578 - 0.925	0.064	0.845 (0.869)	0.298 - 0.931	0.099
Bayesian U-Net	0.935 (0.941)	0.802 - 0.965	0.024	0.838 (0.844)	0.612 - 0.931	0.059	0.834 (0.866)	0.246 - 0.917	0.109
U-NEXtractor	0.249 (0.237)	0.095 - 0.608	0.086	0.259 (0.251)	0.117 - 0.499	0.088	0.259 (0.255)	0.092 - 0.460	0.080
Bayesian U-NEXtractor	0.044 (0.039)	0.016 - 0.169	0.020	0.046 (0.042)	0.019 - 0.106	0.019	0.046 (0.044)	0.014 - 0.114	0.018

4.3 Statistical analysis comparing model performance

Supplementary Table 26 Statistical analysis comparing the performance of the BSN against the MSKCC nomogram, the CAPRA score and the SN on the *test sets* and the *holdout set*. AUC p-values are determined using the fast implementation of DeLong test [17, 18], while CI p-values are calculated with the U-statistics-based C estimator [19]. All other p-values are obtained by bootstrap [20, 21] with 10 000 repetitions. The p-value shown on *test sets* is the median of the p-values calculated on the 5 *test sets*. Color code: increase (cyan), decrease (red) and no significant change (black) of the performance of the BSN compared with the reference model.

	Model	Data	Task																	
			LNI		BCR-FS			MFS			dADT-FS			CRPC-FS			PCSS		
			AUC	BA	CI	CICW	CDA	CI	CICW	CDA	CI	CICW	CDA	CI	CICW	CDA	CI	CICW	CDA
Test sets	MSKCC	CD	1×10^{-1}	3×10^{-1}	8×10^{-1}	5×10^{-1}	1×10^{-1}	—	—	—	—	—	—	—	—	—	5×10^{-3}	3×10^{-2}	3×10^{-2}
Test sets	CAPRA	CD	4×10^{-2}	2×10^{-1}	3×10^{-1}	5×10^{-1}	8×10^{-2}	5×10^{-2}	8×10^{-2}	6×10^{-2}	2×10^{-2}	5×10^{-2}	2×10^{-1}	2×10^{-3}	2×10^{-2}	1×10^{-2}	1×10^{-2}	9×10^{-2}	3×10^{-2}
Test sets	SN	Best	1×10^{-1}	3×10^{-1}	1×10^{-1}	2×10^{-1}	3×10^{-2}	3×10^{-1}	4×10^{-1}	3×10^{-1}	2×10^{-1}	6×10^{-1}	3×10^{-1}	2×10^{-1}	5×10^{-1}	3×10^{-1}	1×10^{-1}	3×10^{-1}	3×10^{-1}
Holdout set	MSKCC	CD	5×10^{-1}	1×10^{-1}	3×10^{-1}	6×10^{-1}	1×10^{0}	—	—	—	—	—	—	—	—	—	3×10^{-1}	6×10^{-1}	3×10^{-2}
Holdout set	CAPRA	CD	4×10^{-1}	1×10^{-1}	3×10^{-1}	2×10^{-1}	3×10^{-1}	1×10^{-1}	4×10^{-1}	2×10^{-2}	7×10^{-1}	6×10^{-1}	7×10^{-1}	9×10^{-1}	6×10^{-1}	3×10^{-1}	6×10^{-3}	2×10^{-1}	5×10^{-1}
Holdout set	SN	Best	6×10^{-1}	1×10^{0}	6×10^{-1}	9×10^{-1}	6×10^{-1}	2×10^{-1}	3×10^{-1}	4×10^{-1}	8×10^{-1}	9×10^{-1}	6×10^{-1}	3×10^{-1}	3×10^{-1}	4×10^{-1}	1×10^{0}	1×10^{0}	1×10^{0}

Supplementary Table 27 Statistical analysis comparing the performance of the MLP with the best data (CD+HCR for LNI, CD+DR for BCR-FS and CD for MFS, dADT-FS, CRPC-FS and PCSS) against the MLP with CD alone, and the SN with the best data. The models are evaluated on the *test sets*. AUC p-values are determined using the fast implementation of DeLong test [17, 18], while CI p-values are calculated with the U-statistics-based C estimator [19]. All other p-values are obtained by bootstrap [20, 21] with 10 000 repetitions. The p-value shown is the median of the p-values calculated on the 5 *test sets*. Color code: increase (cyan), decrease (red), and no significant changes (black) of the performance of the MLP with the best data compared with the reference model.

		Task																
Model	Data	LNI		BCR-FS			MFS			dADT-FS			CRPC-FS			PCSS		
		AUC	BA	CI	CICW	CDA	CI	CICW	CDA	CI	CICW	CDA	CI	CICW	CDA	CI	CICW	CDA
MLP	CD	5×10^{-1}	2×10^{-1}	3×10^{-1}	5×10^{-1}	2×10^{-1}	—	—	—	—	—	—	—	—	—	—	—	—
SN	Best	1×10^{0}	1×10^{0}	8×10^{-1}	5×10^{-1}	8×10^{-1}	3×10^{-1}	5×10^{-1}	5×10^{-1}	3×10^{-1}	5×10^{-1}	1×10^{-1}	5×10^{-1}	7×10^{-1}	2×10^{-1}	4×10^{-1}	6×10^{-1}	3×10^{-1}

5 Illustration of clinical application

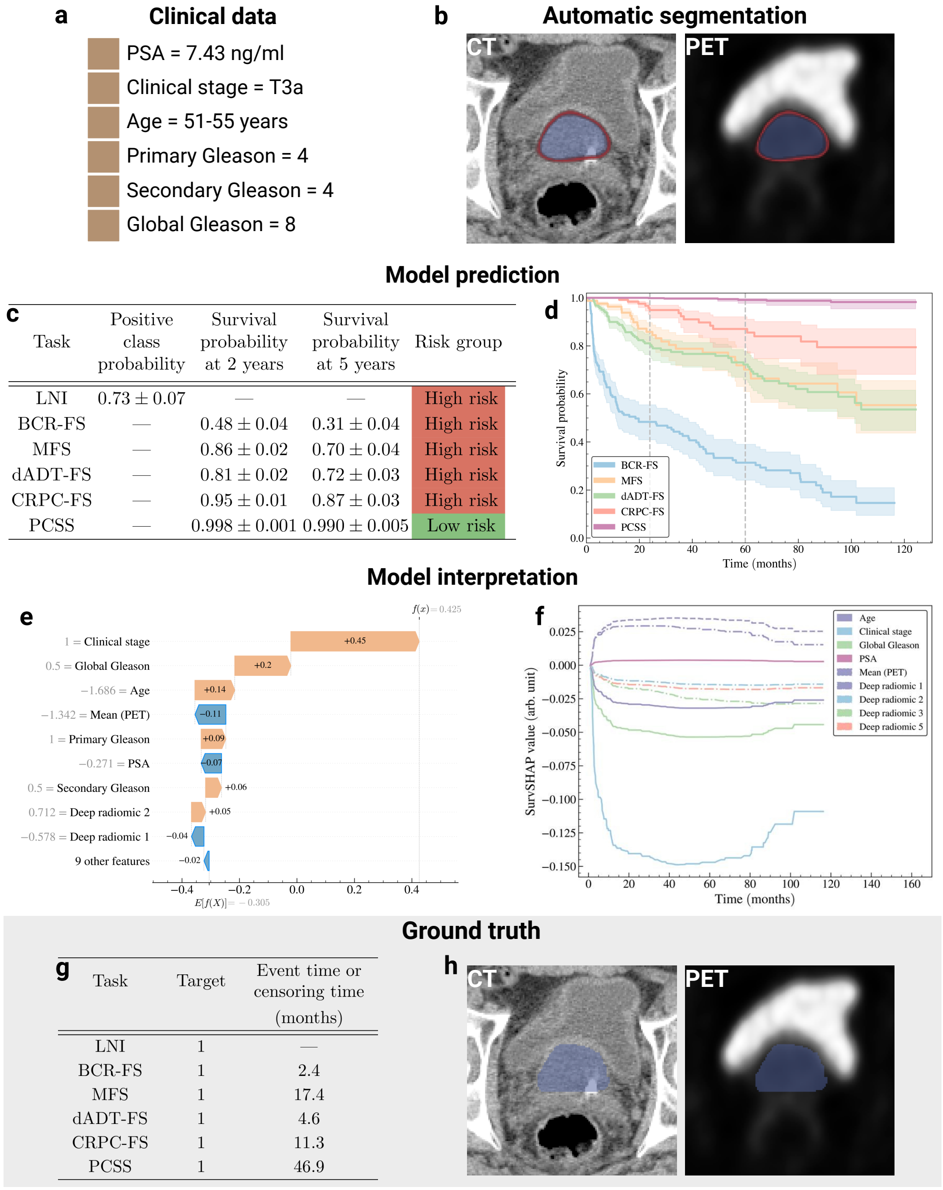

Task	Positive class probability	Survival probability at 2 years	Survival probability at 5 years	Risk group
LNI	0.73 ± 0.07	—	—	High risk
BCR-FS	—	0.48 ± 0.04	0.31 ± 0.04	High risk
MFS	—	0.86 ± 0.02	0.70 ± 0.04	High risk
dADT-FS	—	0.81 ± 0.02	0.72 ± 0.03	High risk
CRPC-FS	—	0.95 ± 0.01	0.87 ± 0.03	High risk
PCSS	—	0.998 ± 0.001	0.990 ± 0.005	Low risk

Task	Target	Event time or censoring time (months)
LNI	1	—
BCR-FS	1	2.4
MFS	1	17.4
dADT-FS	1	4.6
CRPC-FS	1	11.3
PCSS	1	46.9

Supplementary Fig. 20 Prognosis of an arbitrarily selected patient from the *holdout set*. (a) Clinical features of the patient. (b) Segmentation map of the prostate obtained from the Bayesian U-Net trained on the *learning set*. The segmentation map is overlaid on the CT and PET images to illustrate that the region of high FDG uptake by the bladder lies outside the boundaries of the segmentation map. The segmentation map is used to extract handcrafted radiomic features. The DSC between the automatic and the manual segmentation (ground truth) is 0.872. Color code: average prostate segmentation map (blue) and standard deviation (red) over 100 inferences. See Supplementary Fig. Xb for the segmentation map obtained by the Bayesian U-NEXtractor (c) Average prediction and standard deviation of the model over 100 inferences. (d) Average survival curves predicted by the model (line) and 95% confidence interval (shade) over 100 inferences. (e) Shapley additive explanation (SHAP) [22] of the predicted risk of BCR-FS. (f) Time-dependent SHAP (SurvSHAP(t)) [23] of the predicted risk of BCR-FS. (g) Ground truth progression of the patient's cancer. Time represents the survival time when the target value is 1 and acts as a censoring time otherwise. (h) Ground truth prostate segmentation map obtained from manual contouring by a physician. Created in BioRender. Larose, M. (2024) https://BioRender.com/t26d139.

a **Clinical data**

- PSA = 5.10 ng/ml
- Clinical stage = T1-T2
- Age = 56-60 years
- Primary Gleason = 4
- Secondary Gleason = 4
- Global Gleason = 8

b **Automatic segmentation**

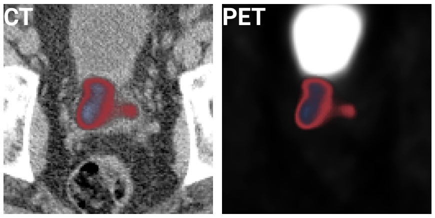

Model prediction

c

Task	Positive class probability	Survival probability at 2 years	Survival probability at 5 years	Risk group
LNI	0.31 ± 0.05	—	—	Low risk
BCR-FS	—	0.62 ± 0.02	0.47 ± 0.03	Low risk
MFS	—	0.924 ± 0.008	0.83 ± 0.02	Low risk
dADT-FS	—	0.85 ± 0.01	0.77 ± 0.02	Low risk
CRPC-FS	—	0.976 ± 0.005	0.94 ± 0.01	Low risk
PCSS	—	1.000 ± 0.001	0.999 ± 0.001	Low risk

d

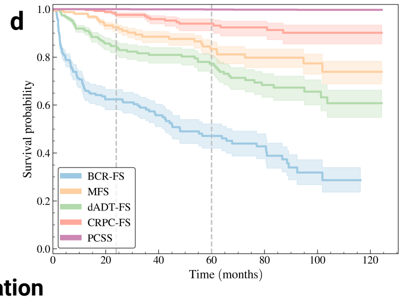

Model interpretation

e

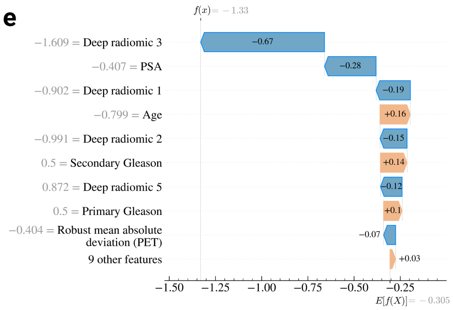

f

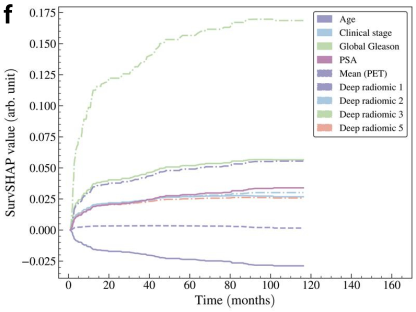

Ground truth

g

Task	Target	Event time or censoring time (months)
LNI	0	—
BCR-FS	0	100.4
MFS	0	100.4
dADT-FS	0	100.4
CRPC-FS	0	100.4
PCSS	0	100.4

h

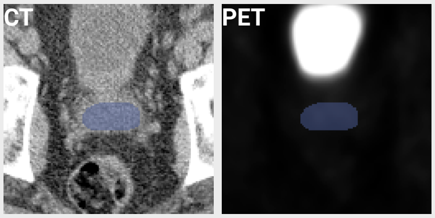

Supplementary Fig. 21 Prognosis of an arbitrarily selected patient from the *holdout set*. (a) Clinical features of the patient. (b) Segmentation map of the prostate obtained from the Bayesian U-Net trained on the *learning set*. The segmentation map is overlaid on the CT and PET images to illustrate that the region of high FDG uptake by the bladder lies outside the boundaries of the segmentation map. The segmentation map is used to extract handcrafted radiomic features. The DSC between the automatic and the manual segmentation (ground truth) is 0.893. Color code: average prostate segmentation map (blue) and standard deviation (red) over 100 inferences. See Supplementary Fig. Xc for the segmentation map obtained by the Bayesian U-NEXtractor.(c) Average prediction and standard deviation of the model over 100 inferences. (d) Average survival curves predicted by the model (line) and 95% confidence interval (shade) over 100 inferences. (e) Shapley additive explanation (SHAP) [22] of the predicted risk of BCR-FS. (f) Time-dependent SHAP (SurvSHAP(t)) [23] of the predicted risk of BCR-FS. (g) Ground truth progression of the patient's cancer. Time represents the survival time when the target value is 1 and acts as a censoring time otherwise. (h) Ground truth prostate segmentation map obtained from manual contouring by a physician. Created in BioRender. Larose, M. (2024) https://BioRender.com/q52p994.

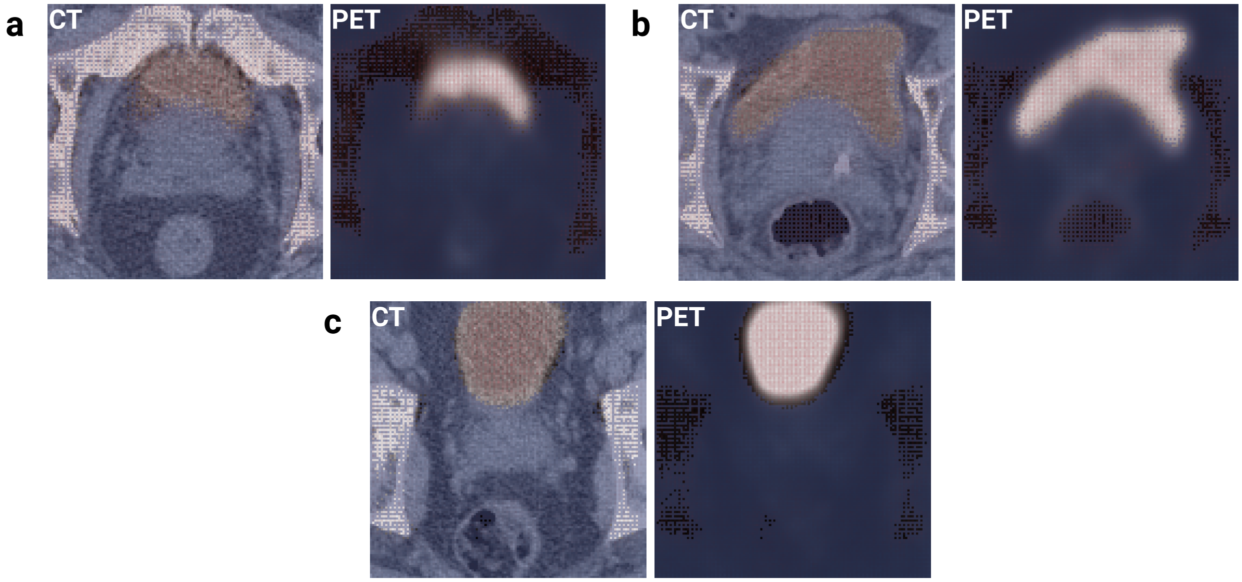

Supplementary Fig. 22 Average prostate segmentation map (blue) and standard deviation (red) over 100 inferences obtained from the Bayesian U-NEXtractor for the patient shown in (a) Fig. 1, (b) Supplementary Fig. 20, and (c) Supplementary Fig. 21. The segmentation map overlaid on the PET image reveals that the Bayesian U-NEXtractor avoids bones and the region of high FDG uptake by the bladder, and segments everything else.

References

[1] Mann, H. B. & Whitney, D. R. On a test of whether one of two random variables is stochastically larger than the other. *The Annals of Mathematical Statistics* **18**, 50–60 (1947).

[2] Cressie, N. & Read, T. R. C. Multinomial goodness-of-fit tests. *Journal of the Royal Statistical Society. Series B (Methodological)* **46**, 440–464 (1984).

[3] Virtanen, P. *et al.* SciPy 1.0: Fundamental Algorithms for Scientific Computing in Python. *Nature Methods* **17**, 261–272 (2020).

[4] Shuster, J. J. Median follow-up in clinical trials. *Journal of Clinical Oncology* **9**, 191–192 (1991). PMID: 1985169.

[5] Schemper, M. & Smith, T. L. A note on quantifying follow-up in studies of failure time. *Controlled Clinical Trials* **17**, 343–346 (1996).

[6] Sathish, N. Let's flip : An approach to understand median follow-up by the reverse kaplan-meier estimator from a statistical programmer's perspective (2019). URL https://www.pharmasug.org/proceedings/2019/ST/PharmaSUG-2019-ST-081.pdf.

[7] Kaplan, E. L. & Meier, P. Nonparametric estimation from incomplete observations. *Journal of the American Statistical Association* **53**, 457–481 (1958).

[8] Borgan, Ø. & Liestøl, K. A note on confidence intervals and bands for the survival function based on transformations. *Scandinavian Journal of Statistics* **17**, 35–41 (1990).

[9] Peto, R. & Peto, J. Asymptotically efficient rank invariant test procedures. *Journal of the Royal Statistical Society. Series A (General)* **135**, 185–207 (1972).

[10] Fleming, T. R. & Harrington, D. P. A class of hypothesis tests for one and two sample censored survival data. *Communications in Statistics-theory and Methods* **10**, 763–794 (1981).

[11] Tierney, J., Stewart, L., Ghersi, G., Burdett, S. & Sydes, M. Practical methods for incorporating summary time-to-event data into meta-analysis. *Trials* **8:**, 16 (2007).

[12] Pölsterl, S. scikit-survival: A library for time-to-event analysis built on top of scikit-learn. *Journal of Machine Learning Research* **21**, 1–6 (2020).

[13] van Griethuysen, J. J. *et al.* Computational Radiomics System to Decode the Radiographic Phenotype. *Cancer Research* **77**, e104–e107 (2017).

[14] Breiman, L. Random forests. *Machine learning* **45**, 5–32 (2001).

[15] Pedregosa, F. *et al.* Scikit-learn: Machine learning in Python. *Journal of Machine Learning Research* **12**, 2825–2830 (2011).

[16] Zou, K. H. *et al.* Statistical validation of image segmentation quality based on a spatial overlap index1: scientific reports. *Academic Radiology* **11**, 178–189 (2004).

[17] DeLong, E. R., DeLong, D. M. & Clarke-Pearson, D. L. Comparing the areas under two or more correlated receiver operating characteristic curves: A nonparametric approach. *Biometrics* **44**, 837–845 (1988).

[18] Sun, X. & Xu, W. Fast implementation of delong's algorithm for comparing the areas under correlated receiver operating characteristic curves. *IEEE Signal Processing Letters* **21**, 1389–1393 (2014).

[19] Kang, L., Chen, W., Petrick, N. A. & Gallas, B. D. Comparing two correlated c indices with right-censored survival outcome: a one-shot nonparametric approach. *Statistics in Medicine* **34**, 685–703 (2015).

[20] Efron, B. Bootstrap Methods: Another Look at the Jackknife. *The Annals of Statistics* **7**, 1 – 26 (1979).

[21] Carpenter, J. & Bithell, J. Bootstrap confidence intervals: when, which, what? a practical guide for medical statisticians. *Statistics in Medicine* **19**, 1141–1164 (2000).

[22] Lundberg, S. M. & Lee, S.-I. in *A unified approach to interpreting model predictions* (eds Guyon, I. *et al.*) *Advances in Neural Information Processing Systems 30* 4765–4774 (Curran Associates, Inc., 2017).

[23] Krzyziński, M., Spytek, M., Baniecki, H. & Biecek, P. Survshap(t): Time-dependent explanations of machine learning survival models. *Knowledge-Based Systems* **262**, 110234 (2023).
